# Subspecific Differentiation Events of Montane Stag Beetles (Coleoptera, Lucanidae) Endemic to Formosa Island

**DOI:** 10.1371/journal.pone.0156600

**Published:** 2016-06-03

**Authors:** Cheng-Lung Tsai, Wen-Bin Yeh

**Affiliations:** Department of Entomology, National Chung Hsing University, Taichung, Taiwan; National Cheng-Kung University, TAIWAN

## Abstract

Taxonomic debates have been carrying on for decades over Formosan stag beetles, which consist of a high proportion of endemic species and subspecies featuring morphological variations associated with local adaptation. With the influence of periodical Pleistocene glaciations and the presence of several mountain ranges, the genetic differentiation and taxonomic recognition, within this medium-size island, of two endemic subspecies for each of four montane stag beetles, i.e. *Lucanus ogakii*, *L*. *kanoi*, *Prismognathus davidis*, and *Neolucanus doro*, has been an appealing issue. Based on monophyletic lineages and population structure, possible divergent scenarios have been proposed to clarify the subspecific status for each of the above mentioned stag beetles. Phylogenetic inferences based on COI+16S rDNA+28S rDNA of 240 Formosan lucanids have confirmed most species are monophyletic groups; and the intraspecific (<2%) and interspecific (>2%) genetic distances of the two mitochondrial genes could be applied concordantly for taxonomic identification. On account of Bayesian-based species delimitation, geographic distribution, population structure, and sequence divergences, the subspecific status for *L*. *ogakii*, *L*. *kanoi*, and *Pri*. *davidis* are congruent with their geographic distribution in this island; and the calibration time based on the mitochondrial genes shows the subspecific split events occurred 0.7–1 million years ago. In addition, a more complicated scenario, i.e. genetic differentiation including introgression/hybridization events, might have occurred among *L*. *ogakii*, *L*. *kanoi*, and *L*. *maculifemoratus*. The geological effects of mountain hindrance accompanied by periodical glaciations could have been vital in leading to the geographical subspecific differentiation of these montane stag beetles.

## Introduction

The family Lucanidae (Coleoptera, Scarabaeoidea) has received much attention because of their remarkable sexual dimorphism, intraspecific variation, and external morphological allometry in males [1–3]. Previous studies on stag beetles showed the intraspecific variation within a species or between subspecies could have been related to their local adaptation, such as larval consumption of dead wood, mating choice of females, and competition for food resources [2–5]. With >1,400 species known throughout the world, stag beetles are particularly abundant in Oriental, Sino-Japanese, and the eastern Palearctic regions [6–9]. East Asia and its adjacent islands have provided ideal geographic topography for species diversification. Species distributing widely with variable morphological characters are suitable for studying their evolutionary history, especially the genetic differentiation between affinity of subspecies [10, 11]. The affinity subspecies within a species is usually recognized according to their geographic distribution and morphological features. For example, several lucanid populations isolated in different islands/regions have been proposed as subspecies for *Dorcus titanus*, *Lucanus maculifemoratus*, and *Neolucanus nitidus* [9]. In general, only one subspecies would be found on an island. Yet, when two subspecies should be recognized, their differentiation processes would be an appealing issue.

Morphological variation of a species is an expression of phenotypic changes in response to diverse topography, climate, and genetic factors throughout its phylogeographic history [12–14]. Within a species, morphologically differentiated populations caused by geographical isolation could be recognized as subspecies by taxonomists. However, the occurrence of gene flow or hybridization among originally isolated subspecies/populations during glaciations might have shaped unique/overlapping morphological characteristics in an organism, which would also complicate taxonomic classification [15]. Thus, the recognition of geographically variable characteristics for closely related species and/or subspecies has sometimes become a challenge for taxonomists [12, 16].

Pleistocene climatic fluctuation has been proposed as a profound factor influencing the origin and diversification of extant organisms [17–19]. Repeated isolations of populations in refugia during Pleistocene glacial cycles have been considered the crucial mode for allopatric speciation in Europe and North America [17–21], though the refugia hypothesis was not considered the major driving force of species origin for neotropical taxa [21–24]. In the refugia hypothesis, isolated populations would accumulate genetic difference through drift and local adaptation over a long period during glaciations [25]. As the interglacial period came, populations would have a chance either to expand their distribution or have secondary contact with other populations [12, 26–28].

Mountainous Taiwan (also known as Formosa), a medium-size island situated in both tropical and subtropical regions in Southeastern Asia, was formed about six million years ago (Mya) by a collision between the Philippine Sea plate and Eurasian plate [29]. A drastic uplift about 3–1 Mya [29, 30] resulted in the appearance of the Central Mountain Range (CMR) extending from northern to southern Taiwan with an altitude up to 3,952 m, together with several branches, i.e. Xueshan Range, Yushan Range, and Alishan Range. These mountain ranges have also been suggested as an important vicariant barrier for the speciation and population differentiation of many organisms, such as fishes, salamanders, toads, crabs, damselflies, and stag beetles during glaciations [12, 28, 31–37]. The most interesting case relates to the recognition of two geographic subspecies for some endemic insects, such as butterflies, dragonflies, damselflies, and stag beetles on this island [12, 34, 38–40].

A total of 52 lucanid species, including 45 endemic species and subspecies, have been identified owing to the specific geographic position and topography of Formosa Island. Several studies of stag beetles in this island have dealt with the taxonomic debates on species or subspecies caused by geographical adaptation and morphological variations affected by Pleistocene glacial cycles and vicariant ranges [12]. Huang and Lin [28] confirmed with molecular and morphological evidences that the three mandible forms in *Lucanus formosanus* were geographic morphs, i.e. northern, central, and southern morphs, instead of genetic differentiation/subspecies. On the other hand, considering several overlapping morphological characteristics, Tsai et al. [12] proposed a single taxon for *Neolucanus swinhoei* complex, which included *N*. *swinhoei*, *N*. *eugeniae*, and *N*. *doro*, with the latter consisting of two subspecies. On an island like Taiwan, the complex speciation processes that a single species with two geographical subspecies would have confronted have become an appealing issue for taxonomists and evolutionary scientists.

**Fig 1:**
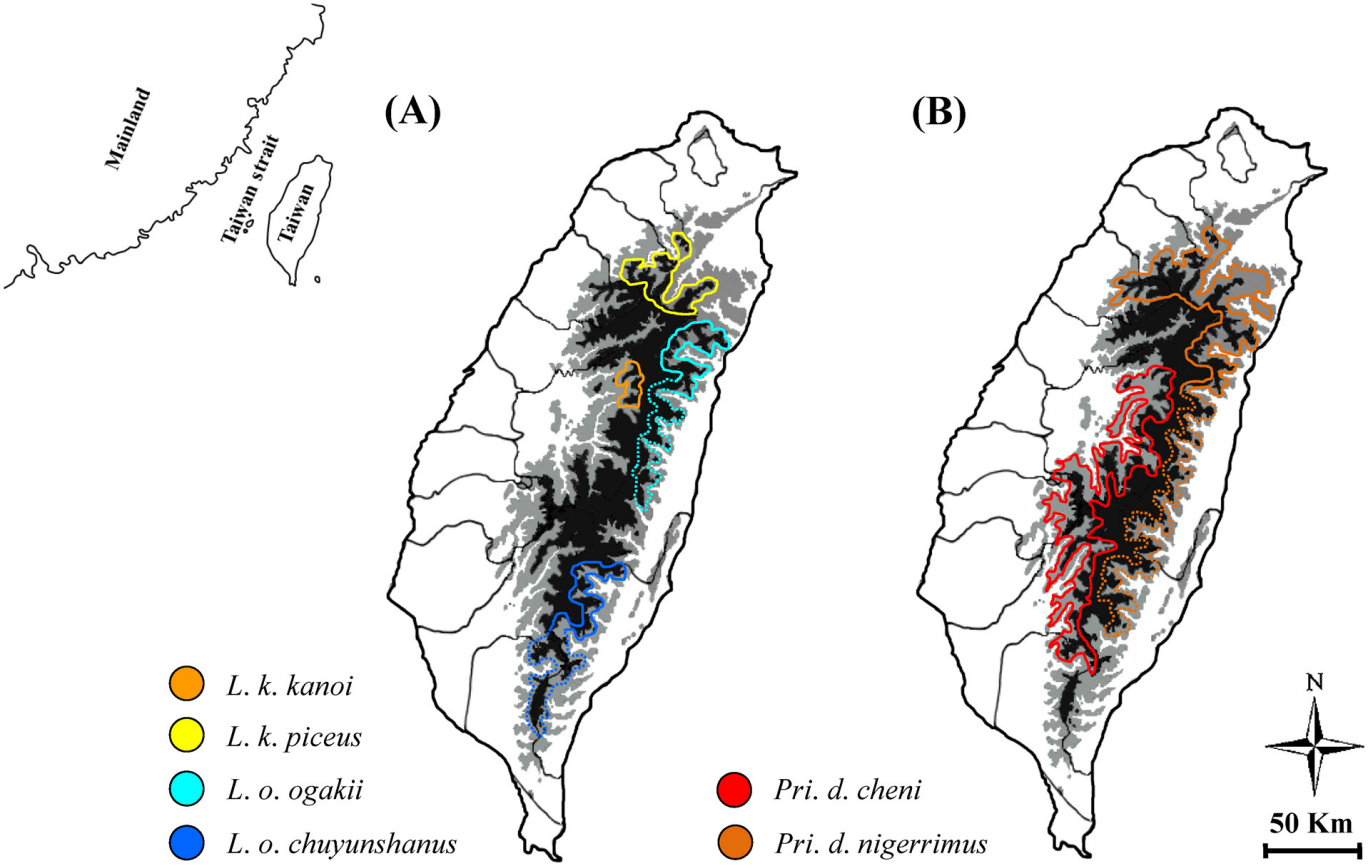
Geographic distribution of the two subspecies for each montane stag beetle. *Lucanus ogakii* and *L*. *kanoi* (A) and *Prismognathus davidis* (B). The solid line shows the distribution region and the dashed line represents the likely distribution area. The mountain range shown in light gray represents an altitude between 1,000 and 2,000 m and dark gray represents an altitude of >2,000 m.

On Formosa Island, three additional stag beetles, i.e. *Lucanus ogakii*, *Lucanus kanoi*, and *Prismognathus davidis*, each having two endemic subspecies, might have faced the same driving forces of mountain hindrance and glacial cycles as mentioned above. *Lucanus ogakii* and *L*. *kanoi* inhabit montane areas ranging from 1,500–2,600 m and 1,000–2,500 m, respectively, on either side of the CMR [41]. *Lucanus ogakii* dwells primarily in eastern Taiwan, with one subspecies *L*. *o*. *ogakii* in the north and another subspecies *L*. *o*. *chuyunshanus* in the south; and the western Taiwan-dwelling *L*. *kanoi* consists of the northern subspecies *L*. *k*. *piceus* and the central subspecies *L*. *k*. *kanoi* with very limited distribution (Fig 1A) [41]. Yet, based on morphological variations of head, clypeus, and male/female genitalia, Huang and Chen [42] treated *L*. *ogakii* as a third subspecies of *L*. *kanoi*. The two endemic subspecies of *Pri*. *davidis* in montane areas ranging from 1,500–2,700 m are *Pri*. *d*. *nigerrimus* in northern/eastern Taiwan and *Pri*. *d*. *cheni* in mid-western/southwestern Taiwan (Fig 1B)[41]. Yet, Huang and Chen [43] revised *Pri*. *d*. *nigerrimus* as a synonym of *Pri*. *d*. *cheni* because the diagnostic character, i.e. darker body color, was variable. Huang and Chen [44] also proposed some additional revisions to the specific synonyms and generic position of stag beetles found on this island. For example, *Dorcus mochizukii* was revised to *Falcicornis tenuecostatus* and a new genus *Miwanus* was set for *D*. *formosanus* and its relevant species. Further evidence would help us to understand their taxonomic status and differentiation history.

For several decades, molecular characteristics have been applied extensively to resolve taxonomic debates and test the divergent time upon species complex, cryptic species, and sibling species. Among the molecular approaches applied extensively to resolve taxonomic debates, a small fragment of commonly used mitochondrial DNA, i.e. cytochrome oxidase subunit I (COI), has been considered efficient in delineating the taxonomic status and relating morphs and developmental life stages in various insects [12, 45–53]. Application of nuclear genes, such as *wingless* or ribosomal DNA region, has been especially helpful in unraveling the hybridization possibility of taxonomically debated species. Moreover, the recently developed methods, e.g. Poisson tree processes (PTP) and General Mixed Yule Coalescent (GMYC) model, based on Bayesian analysis and coalescent framework have also been applied as analytic tools to elucidate the taxonomic status [54, 55]. Since PTP is faster and less requirement for the information of phylogenetic tree than GMYC model, the study herein prefers to use this more convenient and more commonly used approach for the species delineation.

The taxonomical debate in stag beetles has been generally associated with Pleistocene glacial cycles accompanied by vicariant hindrance of mountain ranges in Taiwan. The most interesting issue involves the recognition of two locally distributed subspecies for each of the four montane species mentioned above. The study herein applies molecular data from two mitochondrial genes (COI and 16S rDNA) and two nuclear genes (28S rDNA and *wingless*) to depict the genetic variation in 262 individuals of 48 lucanid species and subspecies to clarify the status of the taxonomically debated stag beetles. Based on the monophyletic lineages, geographical distribution, population structure, and species delimitation such as PTP, we further address the subspecific status and the possible hybridization events between the two subspecies in each of *L*. *ogakii*, *L*. *kanoi*, *Pri*. *davidis*, and *N*. *doro*. Hypotheses on the subspecific divergent scenarios are proposed: (1) populations/subspecies displaying variable morphological characteristics, which might be due to local adaption, have a similar genetic composition, such as the mandible morphs in *L*. *formosanus*; (2) morphologically differentiated subspecies may represent divergent lineages in congruence with their discontinuous distribution; and (3) further genetic differentiation involves introgression/ hybridization events, such as in *N*. *swinhoei* complex.

## Materials and Methods

### Sample collection

Forty-eight species and subspecies of stag beetles belonging to 14 genera collected from Taiwan and its neighboring islands were preserved in 95% EtOH at -20°C for molecular analyses (S1 Table). At least three individuals were analyzed for each species. Six species from closely related families of Lucanidae within the same superfamily Scarabaeoidea including Geotrupidae (*Phelotrupes formosanus*), Passalidae (*Aceraius grandis* and *Leptaulax formosanus*), and Scarabaeidae (*Allomyrina dichotoma tunobosonis*, *Anomala aulacoides*, and *Xylotrupes mniszechi tonkinensis*), were used for outgroup comparison in phylogenetic analyses. All voucher specimens are deposited in Department of Entomology, National Chung Hsing University, Taichung, Taiwan.

### Ethics statement

We confirm no endangered or protected species of stag beetle was involved in this study. All field collections in protected areas, i.e. national parks and national forested land, were permitted by the authorities. In the National Park, the collection permission was issued by the Headquarters of Yangmingshan National Park (permission number 20140101), Sheipa National Park (permission numbers 1030001234, 1030000674), and Taroko National Park (permission numbers 201103020129, 201202200200, 201303080267). Field collection in each national forested land was authorized by the Forestry Bureau: the collection permission was issued by the Headquarter of Hsinchu Forest District Office (permission numbers 1022102680, 1032102837), Dongshih Forest District Office (permission numbers 1023161059, 1033161025), Nantou Forest District Office (permission numbers 1024161154, 1034161079), Chiayi Forest District Office (permission numbers 1025161568, 1035161308), Pingtung Forest District Office (permission numbers 1026161180, 1036161438), Luodong Forest District Office (permission numbers 1021102104, 1031151311), Hualien Forest District Office (permission numbers 1028161017, 1038160848), and Taitung Forest District Office (permission number 1027240244), respectively.

### DNA extraction, amplification, and sequencing

Genomic DNA was extracted from metacoxa muscle using a QuickExtract DNA extraction kit (Epicentre Biotechnologies, Madison, WI) with the protocol by Tsai et al. [12]. The primer sets used to amplify two mitochondrial genes, i.e. COI and 16S rDNA, and the nuclear gene 28S rDNA are listed in Table 1. Moreover, additional primer pairs of the nuclear gene *wingless* were applied for taxonomically debated taxa in the genus *Lucanus* and *Prismognathus*. The PCR was conducted in a volume of 25 μl and the programming conditions were 94°C for 2 min as the initial denaturation, 35 cycles of 94°C for 40 s, 45–52°C for 40 s, and 72°C for 40 s, then 72°C for 10 min as a final extension. PCR products were purified from 1% agarose gel with QIA quick Gel Extraction Kit (Qiagen, Hilden, Germany) and then sequenced using a Taq dye terminator Cycle Sequencing Kit (Applied Biosystems, Foster City, CA) and an ABI 377A sequencer. All sequences were deposited in GenBank under the inclusive following accession numbers (COI: LC074471–LC074690, LC091038–LC091039; 16S rDNA: LC074974–LC075188, LC091040–LC091041; 28S rDNA: LC066683–LC066936, LC126100–LC126101); *wingless*: LC077663–LC077693, LC126084–LC126099) (S1 Table).

**Table 1:** Primers and their amplification size of each amplicon in this study.

Gene	Primer	Sequence 5'→3'	Size (bp)	References
COI	CI-46Coleoptera (+)	AACCATAAAGATATTGGAAC	686	Tsai et al. [12]
	CI-731Coleoptera (-)	CCAAAAAATCAAAATAAATGTTG		
	COI-68_Dorcus_F (+)	TATAYTTTCTTYTAGGAAGRTG	664	In this study
	COI-77_Cyclo_F (+)	TYCTTGGAAGATGATCAGGWAT	655	
	COI-731Lucanidae (-)	CCRAARAATCARAAHAARTGYTG		
16S rDNA	16SR21 (+)	GCCTGTTTATCAAAAACAT	550	Yeh et al. [56]
	16S22 (-)	CCGGTCTGAACTCAGATCA		
28S rDNA	28Sa (+)	TCCGTAACTTCGGAACAAGGATT	700	Lin et al. [57]
	28Sb (-)	TGTACCGCCCCAGTCAAACT		
	28SA1 (+)	CCGTCTTGAAACACGGACCAAG	700	Li et al. [58]
	28SB1 (-)	TTCGGCAGGTGAGTTGTTACACAC		
*wingless*	Wg_Luc_1_F (+)	GAAGRCCTGYTGGATGAGGCTT	441	In this study
	Lucanus_wg2a (-)	TTGCACCTTTCGACGATGGCGATCTC		Lin et al. [59]
	Wg550F (+)	ATGCGTCAGGARTGYAARTGYCAYGGYATGTC		Wild and Maddison [60]
	WgAbRZ (-)	CACTTNACYTCRCARCACCARTG		
	Wg578F (+)	TGCACNGTGAARACYTGCTGGATG	476	Ward and Downie [61]
	WgAbR (-)	ACYTCGCAGCACCARTGGAA		Abouheif and Wray [62]

“+” and “-” are upstream and downstream primers, respectively.

### Phylogenetic analyses

Sequences were piled up by Bioedit 7.0 [63] and then aligned using Muscle Multiple Alignment option in SeaView4 [64]. Genetic divergences among taxa were analyzed using MEGA 6.0 via p-distance [65]. DNA sequences COI (a total of 148), 16S rDNA (131), and 28S rDNA (64) of Lucanidae, were downloaded from NCBI for genetic distance analyses (S1 Table).

Divergence congruence among genes of COI, 16S rDNA, and 28S rDNA was examined before they were joined in phylogenetic reconstruction. Each gene was converted to p-distance data matrix and the analysis was carried out in R [66] using congruence among genetic distance matrices (CADM) [67] via APE 3.4 [68]. Three partitioned genes, i.e. COI, 16S rDNA, and 28S rDNA, were concatenated to conduct the phylogenetic inferences on the basis of maximum likelihood (ML) and Bayesian inference (BI). For ML, GTR+I+G was selected as the preferred substitution model in RAxML v. 8.2.4 [69]. The best-scoring ML was conducted from 200 replications as suggested by the manual, each starting with a randomized parsimony tree. Then, the support of nodes was examined by 100 nonparametric bootstraps. As to BI, the best-fit substitution models for COI, 16S rDNA, and 28S rDNA were analyzed in jModelTest 0.1 [70] using Bayesian Information Criterion (BIC), and the best-fit ones were TIM1+I+G, TIM1+I+G, and K80+I+G, respectively. Three partitioned genes analyzed for BIs were performed in MrBayes v. 3.2 [71] with three heat chains and one cold chain, and conducted for 1 × 10^7^ generations with sampling every 100 generations. The analysis was finished dependent on the average standard deviation of split frequencies less than 0.01. The first 25% of trees were discarded as burn-in, and the remaining 75% of trees were utilized to reconstruct a consensus tree.

In addition, the nuclear gene *wingless* was also exploited herein to conduct a ML tree for each of the taxonomically debated species of *Lucanus* and *Prismognathus* to address the putative hybridization of these beetles.

### Diversification calibration

The diversification time for taxonomically debated taxa was analyzed using a strict molecular clock in BEAST v. 1.6.1 [72]. The best-fit model employed in BEAST was determined by jModelTest 0.1 [70] using Bayesian Information Criterion (BIC). For *Prismognathus*, the best-fit models for both COI and 16S rDNA were HKY+G; and for *L*. *ogakii*, *L*. *kanoi*, and *L*. *maculifemoratus*, and HKY+I+G and TrN+ I were used. The substitution rates for these stag beetles were estimated using 1.77%/lineage per million years (Myr) for COI and 0.53%/lineage/Myr for 16S rDNA, calibration data from other beetles [73]. A strict molecular clock was applied in MCMC running for 1 × 10^8^ generations with samplings every 1 × 10^4^ generations. The output results of the related parameter values and Effective Sample Size (ESS) for posterior distribution were analyzed in Tracer v. 1.5 [74]. The analysis was performed until there was no warning message with the suggested value; then the initial 10% of the run was discarded as burn-in.

### Species delimitation

To delineate the species boundary for taxonomically debated taxa, the recognition of species were analyzed via *BEAST and PTP using multilocus data, i.e. COI, 16S rDNA, 28S rDNA, and *wingless*. For comparison, *L*. *kurosawai*, *L*. *k*. *kanoi*, and *N*. *nitidus* were selected as outgroups for lineages of *Lucanus*, *Prismognathus*, and *Neolucanus*, respectively. With no genetic variation found, 28S rDNA was not included for *N*. *swinhoei* and *Prismognathus* delineation.

A sequence-based coalescent *BEAST [75], on the basis of posterior probability, implemented in BEAST v. 1.6.1 was used to reconstruct the species tree for each taxonomically debated taxa. For different partitioned genes, the priors were set for clock model as a strict clock, speciation tree prior to the Yule Process, and population size model as a Piecewise constant Population Size Model. The analysis was run for 1 × 10^8^ MCMC generations with samplings every 1 × 10^4^ generations. The output results were analyzed in Tracer v. 1.5 [74] until there was no warning message with the suggested value; then the initial 10% of the run was discarded as burn-in. For PTP [76], the analyses were performed on a bPTP server (http://species.h-its.org/ptp/) using ML topology reconstructed by RAxML. Bayesian supported (BS) values on nodes were regarded as the species confidence. The analyses were run for 300,000 MCMC generations, with the thinning being set to 100 and burn-in at 10%.

### Network analyses

To unravel the diversified processes of haplotypes of the taxonomically debated *L*. *ogakii*, *L*. *kanoi*, *L*. *maculifemoratus*, and *Prismognathus*, haplotype networks for COI and 16S rDNA were analyzed using the program TCS v. 1.21 with a 95% connection limitation [77], and the indel was regarded as 5th state in 16S rDNA.

### Hybridization test for taxonomically debated species

To detect possible hybridization among taxonomically debated stag beetles, a model-based Bayesian clustering software STRUCTURE v 2.3.4 [78] was applied using the admixture model and the correlated allele frequency between populations [79]. The number of possible cluster (K) was set on the basis of possible clusters from 1 to 5, and a total of 15 runs were performed for each K with a 50,000 burn-in followed by 100,000 MCMC replications. The usage of the K value was determined on the basis of ΔK which was estimated by the Evanno method [80] using the Structure Harvester software online website (http://taylor0.biology.ucla.edu/structureHarvester/#).

## Results

### Sequence composition of COI, 16S rDNA, and 28S rDNA genes in Lucanidae

COI, 16S rDNA, and 28S rDNA were successfully amplified for 240 stag beetles of 48 species and subspecies in 14 genera. The fragment sizes for COI, 16S rDNA, and 28S rDNA are 610 bp, 512 bp, and 620 bp, respectively. The average base compositions for G, A, T, C are 16.8%, 28.5%, 32.3%, 22.4% in COI gene, and 20.5%, 30.8%, 39.1%, 9.6% in 16S rDNA, and 31.0%, 25.2%, 18.9%, 24.9% in 28S rDNA, respectively.

### Sequence variations in Lucanidae

**Fig 2:**
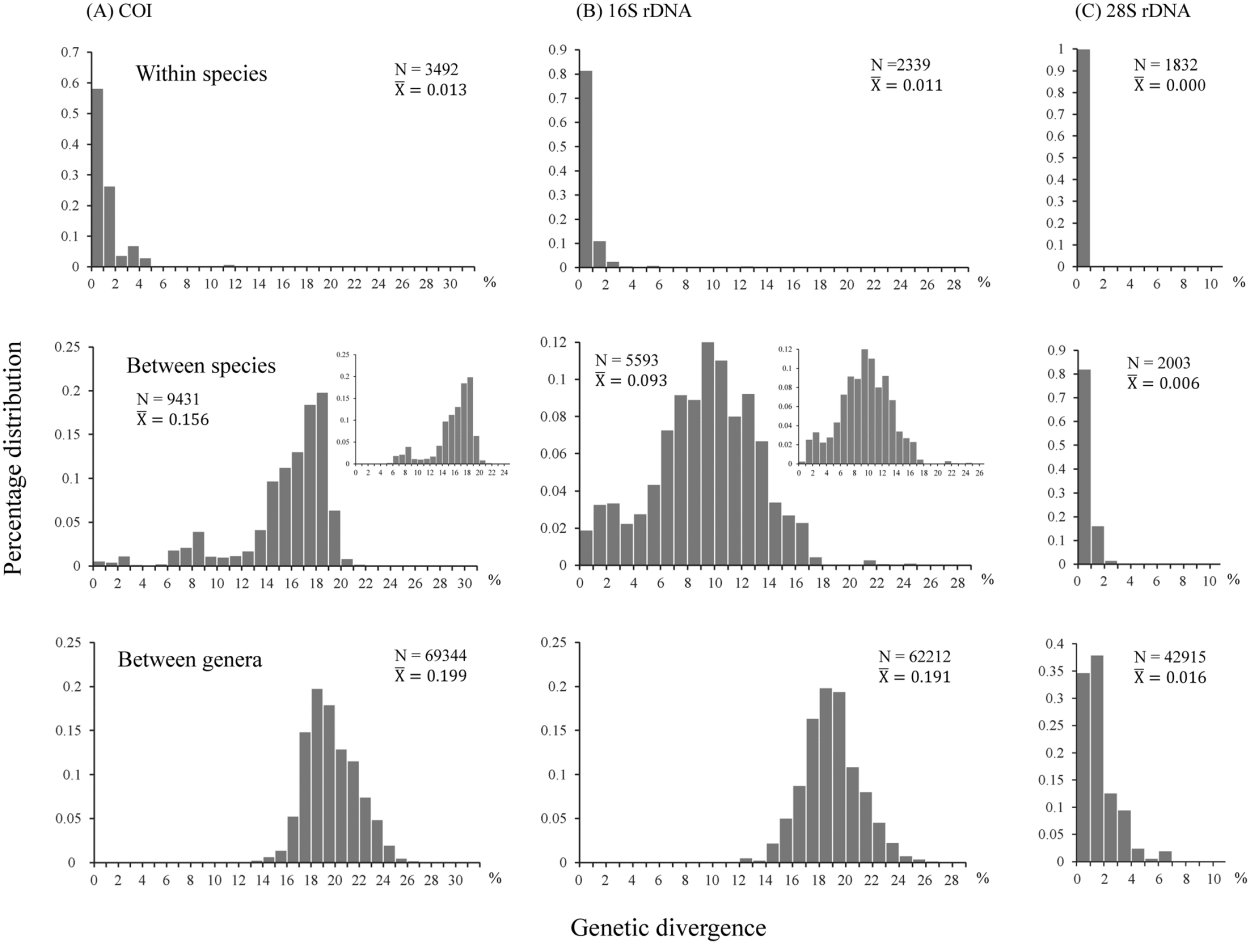
Genetic variations (p-distance) of COI (A), 16S rDNA (B), and 28S rDNA (C) in Lucanidae. Nucleotide divergence of pairwise comparisons for individuals within species (upper), for species within genera (middle), and among genera of Lucanidae (bottom) are shown. Relevant data excluding those of taxonomically debated species are shown in the upper right panel.

Fig 2 shows the uncorrected nucleotide divergence and frequency distribution of the pairwise sequence difference for each of COI, 16S rDNA, and 28S rDNA in three categories: 1) among individuals within species at 0–5%, 0–3%, and 0–1%, respectively; 2) among species of a given genus at 6–20%, 0–18%, and 0–2%, respectively; and 3) among genera in Lucanidae at 14–25%, 15–24%, and 0–6%, respectively. While the nucleotide divergence for intraspecies is <2% in COI and 16S rDNA, and the genetic distances of the interspecies and intergenera are overlapping (Fig 2A and 2B). Similarly, <2% nucleotide divergence of the conserved 28S rDNA, is observed for intra- and interspecies (Fig 2C); and no genetic variation in 28S rDNA has been detected in many congeneric species.

**Fig 3:**
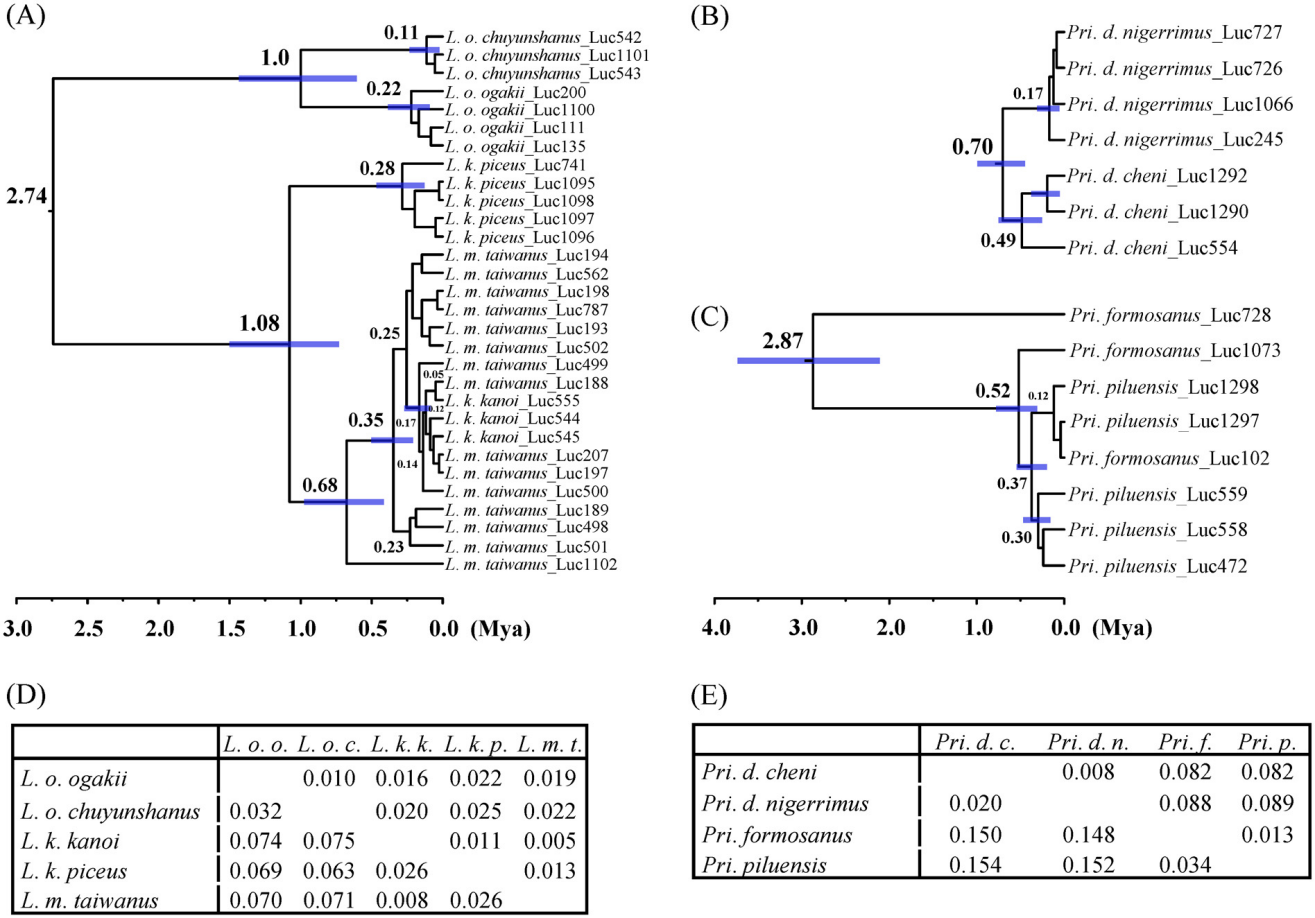
Divergence time estimation and pairwise divergences based on COI and 16S rDNA for taxonomically debated stag beetles. Calibration dating based on COI+16S rDNA for (A) *Lucanus ogakii*, *L*. *kanoi*, and *L*. *maculifemoratus taiwanus*, (B) *Prismognathus davidis nigerrimus* and *Pri*. *d*. *cheni*, and (C) *Pri*. *piluensis* and *Pri*. *formosanus*. Interspecific pairwise comparison using p-distance for five *Lucanus* taxa (D) and four *Prismognathus* taxa (E) are shown. COI divergences: lower-left, and 16S rDNA divergences: upper-right (D, E).

The subspecies of three montane stag beetles examined herein have distinct genetic variations in both COI and 16S rDNA. The average divergences of COI for *L*. *o*. *ogakii* vs. *L*. *o*. *chuyunshanus*, *L*. *k*. *kanoi* vs. *L*. *k*. *piceus*, and *Pri*. *d*. *cheni* vs. *Pri*. *d*. *nigerrimus* are 3.2%, 2.6%, and 2%; and those of 16S rDNA are 1.0%, 1.1%, and 0.8%, respectively (Fig 3D and 3E). However, intraspecific and interspecific genetic variations of both genes are overlapping for *L*. *k*. *kanoi* and *L*. *m*. *taiwanus*, with an average genetic distances of 0.8% and 0.5% for COI and 16S rDNA, respectively (Fig 3D). Yet, much higher genetic distances for these genes, i.e. 2.8% and 0.8%, have been observed within *L*. *m*. *taiwanus*. If the latter and other genetic admixture species were excluded, an overlapping of genetic distances between interspecies and intraspecies was only observed in 16S rDNA (Fig 2A and 2B, upper right).

### Phylogenetic analyses

**Fig 4:**
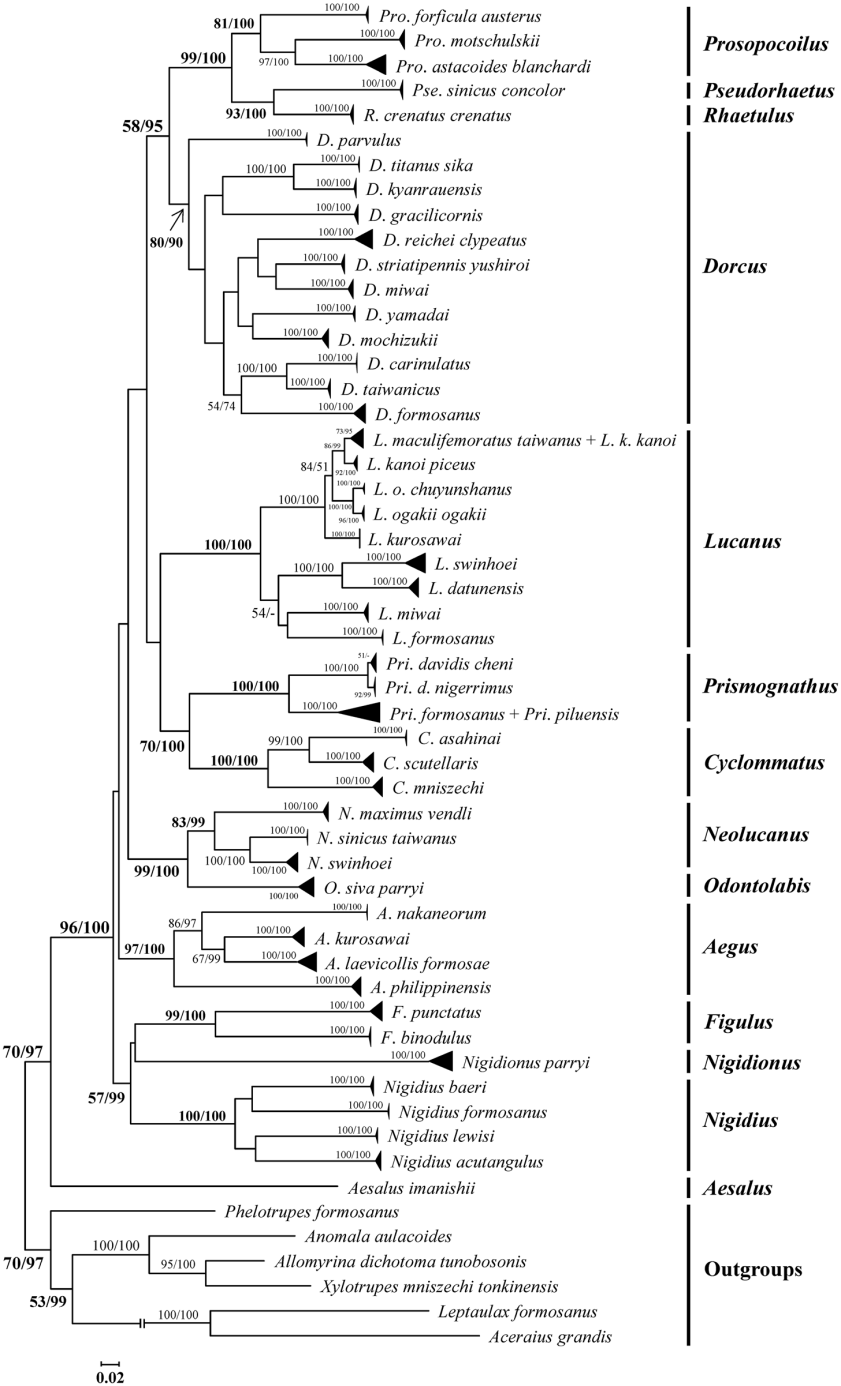
Phylogenetic inferences based on COI+16S rDNA+28S rDNA using maximum likelihood (ML). Both bootstrapping values of ML (left) and posterior probabilities of Bayesian inference (BI) (right) >50% are shown at nodes.

The congruence among COI, 16S rDNA, and 28S rDNA was confirmed using R software with significant statistical support values (Mantel correlation: COI vs. 16S rDNA = 0.56, p < 0.001; COI vs. 28S rDNA = 0.65, p < 0.001; 16S rDNA vs. 28S rDNA = 0.50, p < 0.001). Phylogenetic inference based on COI+16S rDNA+28S rDNA using maximum likelihood (ML) reveals each genus studied herein formed a well-defined monophyletic group (Fig 4). Phylogenetic analysis also shows all species, except *L*. *kanoi/L*. *maculifemoratus* and *Pri*. *formosanus/Pri*. *piluensis*, are monophyly.

### Species delimitation and possible hybridization of taxonomically debated *Lucanus*, *Prismognathus*, and *Neolucanus* stag beetles

**Fig 5:**
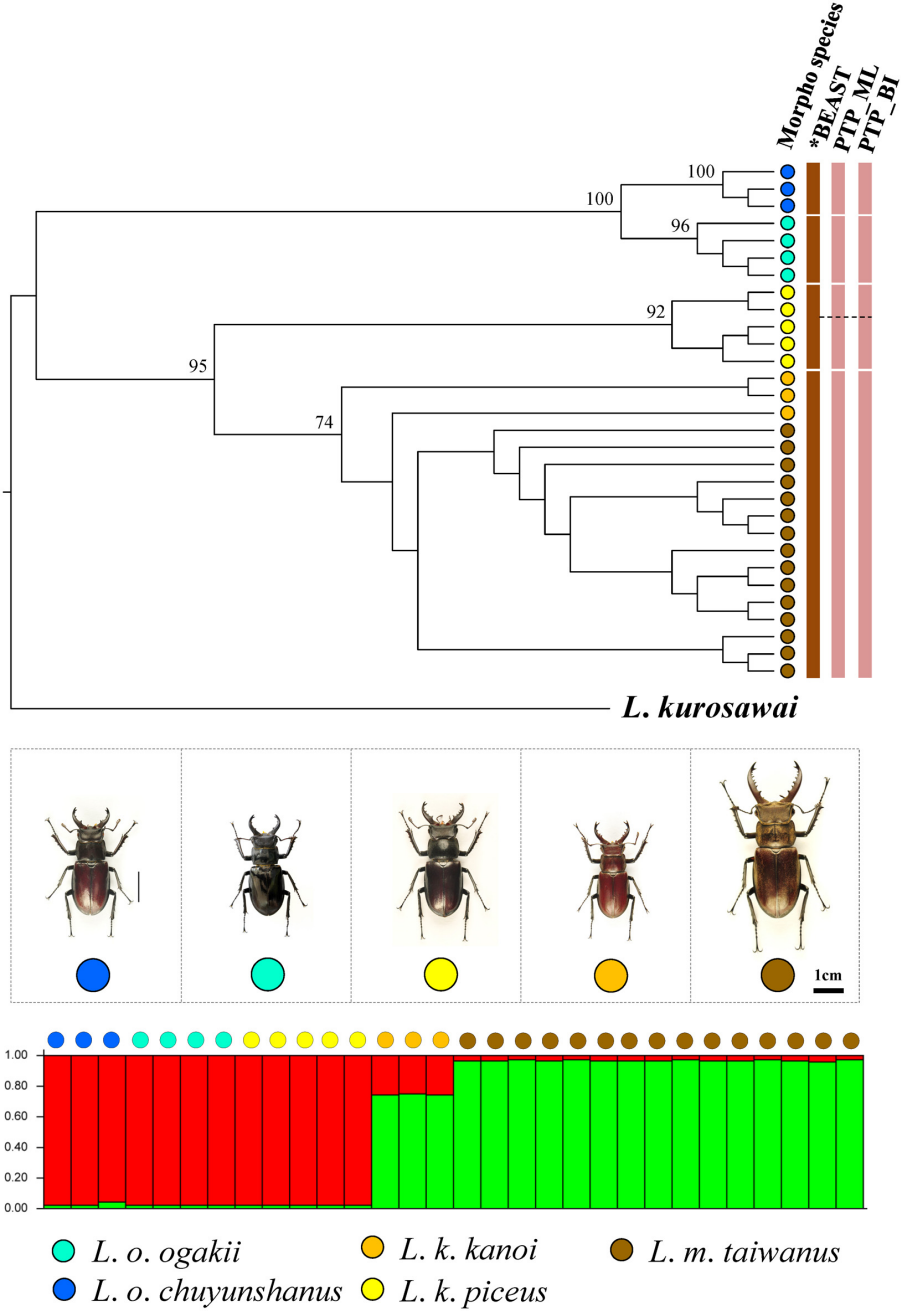
Multilocus species delimitation and hybridization test of the five *Lucanus* taxa. The species delimitations suggested by *BEAST and PTP are shown at the right side of the phylogram. STRUCTURE analysis assumed the optimal Bayesian clustering (K = 2) of the addressed five taxa. Each bar stands for a single individual. Morphological taxa are represented by different colors. The two groups found in *L*. *k*. *piceus* by PTP are represented by a dashed line.

**Fig 6:**
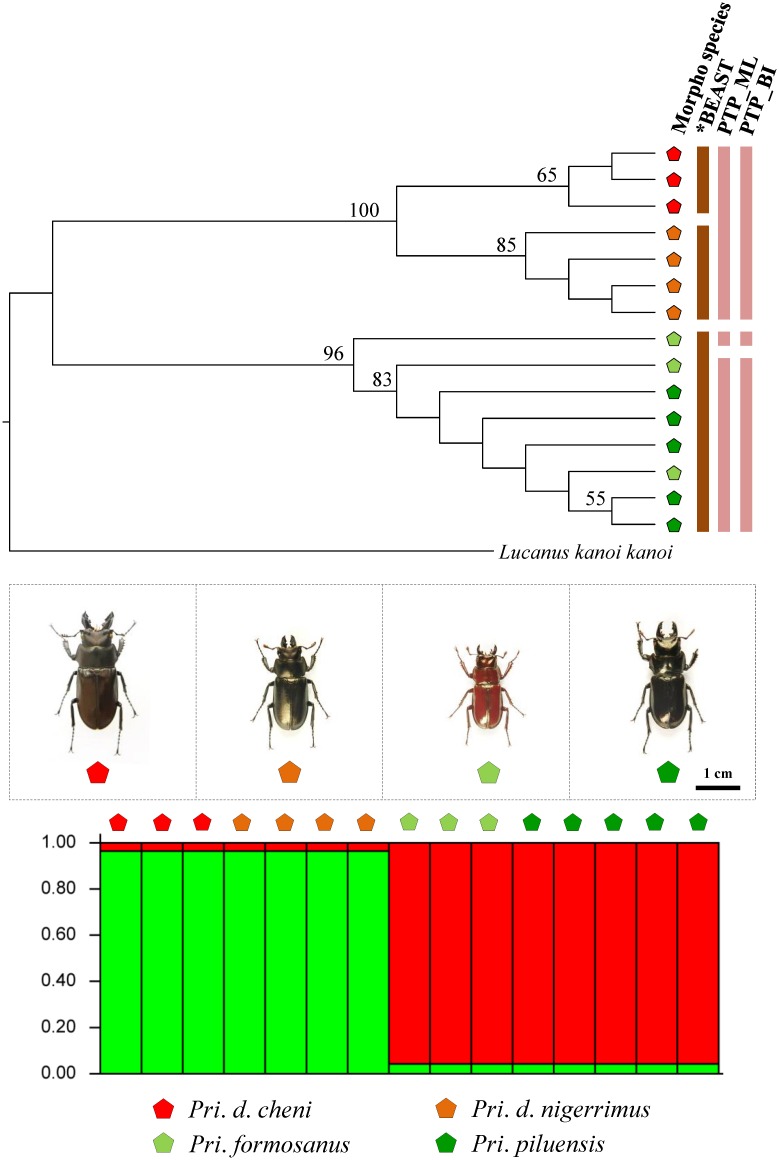
Multilocus species delimitation and hybridization test of the four *Prismognathus* taxa. The species delimitations recommended by *BEAST and PTP are shown at the right side of the phylogram. STRUCTURE analysis assumed the optimal Bayesian clustering (K = 2) of the four taxa. Each bar stands for one individual. Morphological taxa are represented by different colors.

**Fig 7:**
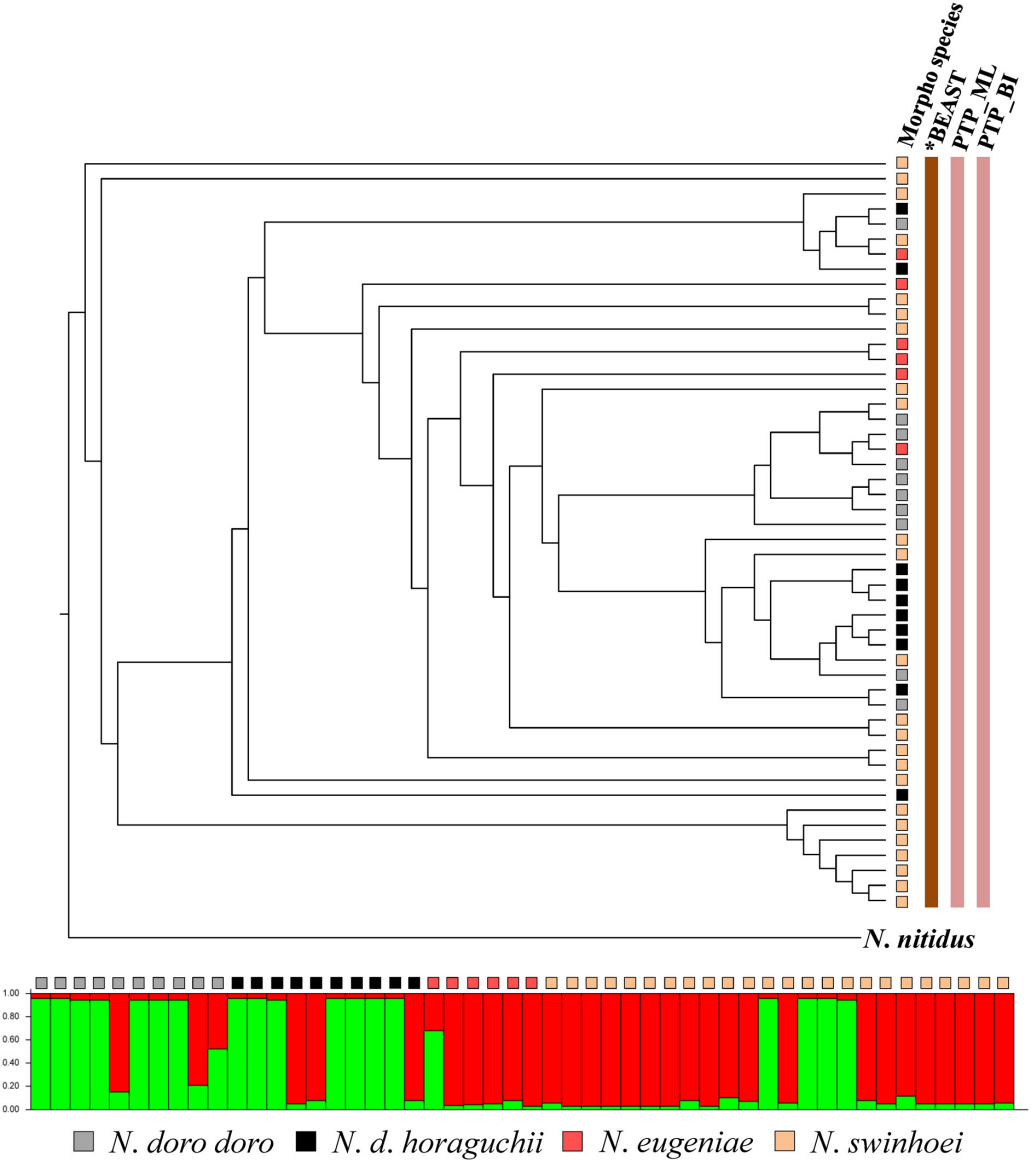
Multilocus species delimitation and hybridization test of the four *Neolucanus* taxa. The species delimitations recommended by *BEAST and PTP are shown at the right side of the phylogram. STRUCTURE analysis assumed the optimal Bayesian clustering (K = 2) of the addressed taxa. Each bar stands for one individual. Morphological taxa are represented by different colors.

Data obtained from multilocus species delimitation and model-based clustering simulation are able to provide reliable information for taxonomic treatment. For *Lucanus*, although two clusters were identified for the five known morphological taxa by STRUCTURE analysis, the species delimitation analyses recognized four groups, i.e. *L*. *o*. *ogakii*, *L*. *o*. *chuyunshanus*, *L*. *k*. *piceus*, and *L*. *maculifemoratus taiwanus* (including *L*. *k*. *kanoi*) (Fig 5). In *Prismognathus*, STRUCTURE analysis has shown *Pri*. *davidis* forms a cluster separated from *Pri*. *formosanus* plus *Pri*. *piluensis*, and species delimitation analysis of *BEAST revealed *Pri*. *davidis* has two well defined subspecies. Further, these analyses also suggested *Pri*. *formosanus* and *Pri*. *piluensis* should belong to a single taxon (Fig 6). For *N*. *swinhoei*, while STRUCTURE analysis showed two optimal clusters for the four known morphological taxa, both *BEAST and PTP indicated a single cluster (Fig 7) as proposed by Tsai et al. [12].

### Genetic differentiation in taxonomically debated *Lucanus* and *Prismognathus* stag beetles

**Fig 8:**
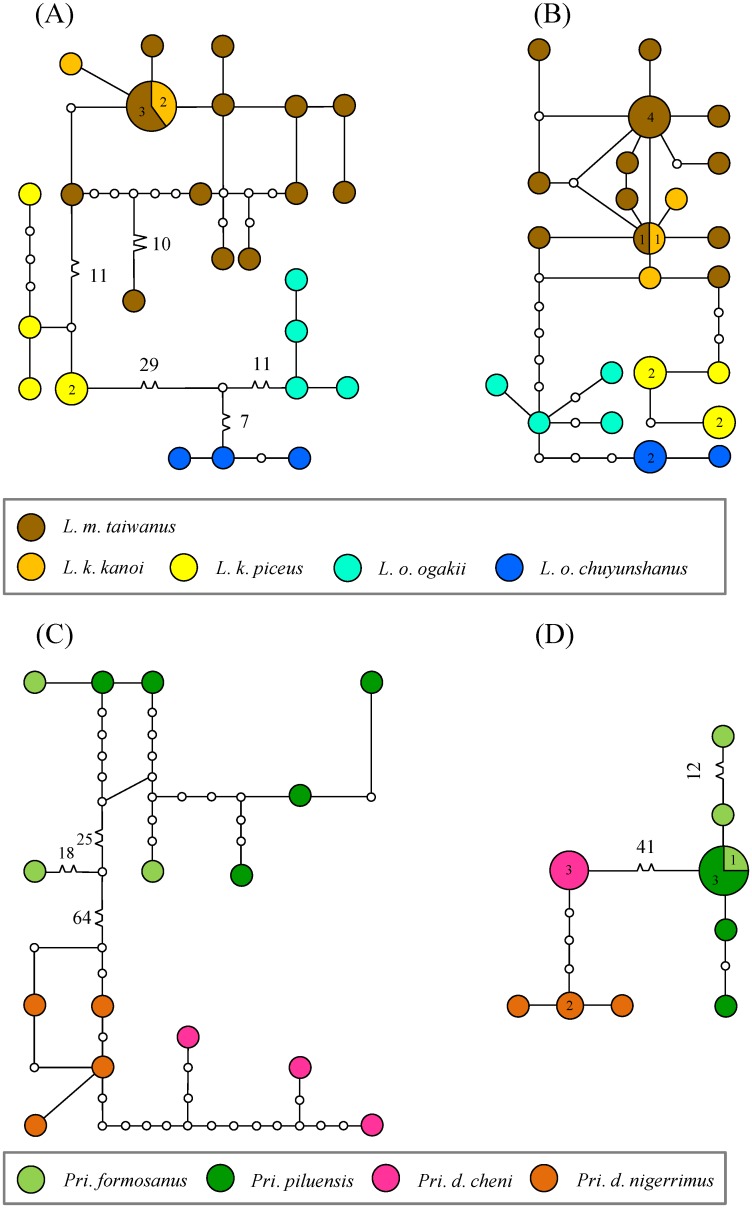
Haplotype networks of taxonomically debated *Lucanus* and *Prismognathus* stag beetles based on mitochondrial COI (A, C) and 16S rDNA (B, D). Taxa are represented by different colors. With the smallest circle standing for one individual, the number of individuals for each haplotype is shown in the circle.

Statistical parsimony networks of COI and 16S rDNA were used to examine the haplotypes evolving pattern in the taxonomically debated taxa, i.e. *L*. *ogakii*, *L*. *kanoi*, and *L*. *maculifemoratus*; and *Pri*. *formosanus*, *Pri*. *piluensis*, and *Pri*. *davidis* (Fig 8). High haplotype diversity exists in these stag beetles, especially in *L*. *maculifemoratus* (Fig 8A and 8B). The substitution steps of *L*. *kanoi* and *L*. *maculifemoratus* from their sister group *L*. *ogakii* are at least 36 and 6 steps for COI and 16S rDNA, respectively (Fig 8A and 8B). The haplotype networks indicate *L*. *k*. *piceus* forms a group of its own, and yet, its sibling *L*. *k*. *kanoi* is unexpectedly close to and shares the haplotype with *L*. *maculifemoratus*. For *Prismognathus*, each of the two subspecies of *Pri*. *davidis* forms its own group in COI and 16S rDNA (Fig 8C and 8D). Though with highly diversified haplotypes, the congeneric *Pri*. *formosanus* and *Pri*. *piluensis* showed an admixed pattern (Fig 8C and 8D).

Maximum likelihood (ML) phylogenetic trees based on the nuclear gene *wingless* used to address the hybridization possibility showed all five *Lucanus* taxa, including the outgroup *L*. *ogakii*, are non-monophyletic (S1 Fig). In addition, considerable heterogeneity, eleven nucleotide positions out of 441 bp in the *wingless* gene, was observed (S1 Fig and S2 Table). In *Prismognathus*, two major lineages, i.e. *Pri*. *davidis* and *Pri*. *piluensis* plus *Pri*. *formosanus*, were found, with two heterogeneous nucleotide positions out of 433 bp for each lineage (S3 Table).

### Divergence calibration in taxonomically debated *Lucanus* and *Prismognathus* stag beetles

Calibration time in these taxonomically debated species was analyzed based on COI and 16S rDNA genes to delineate their possible differentiation histories. It shows the split events in the subspecies of *L*. *ogakii* and *L*. *kanoi* occurred ca. 0.7–1 Mya (Fig 3A). Among the five taxa of *L*. *ogakii*, *L*. *kanoi*, and *L*. *maculifemoratus*, the two major lineages *L*. *ogakii* and *L*. *maculifemoratus*/*L*. *kanoi* diverged ca. 2.7 Mya (Fig 3A). At approximately 1 Mya, the *L*. *ogakii* lineage diverged into two subspecific lineages, namely, *L*. *o*. *ogakii* and *L*. *o*. *chuyunshanus*; and *L*. *k*. *piceus* diverged from the *L*. *k*. *kanoi* lineage. Hybridization between *L*. *m*. *taiwanus* and *L*. *k*. *kanoi* very likely occurred 0.05–0.12 Mya during the recent Würm glaciations. In the genus *Prismognathus*, two subspecies of *Pri*. *davidis*, i.e. *Pri*. *d*. *cheni* and *Pri*. *d*. *nigerrimus*, diverged ca. 0.7 Mya in the middle Pleistocene (Fig 3B); and *Pri*. *formosanus* and *Pri*. *piluensis* show an origin ca. 2.8Mya (Fig 3C).

## Discussion

### Genetic divergence and phylogeographic history for montane stag beetles

This study revealed the taxonomically debated stag beetles, i.e. *L*. *ogakii*, *L*. *kanoi*, and *Pri*. *davidis*, could have confronted and evolved under similar geological events as proposed for some other organisms on Formosa Island. Morphological variations between populations/subspecies or species in montane stag beetles might be taken as an expression responding to diverse topography and Pleistocene glaciations throughout their phylogeographic history. A restricted habitat, i.e. refugia, formed repeatedly during Pleistocene glacial cycles, considered as the crucial mode for allopatric speciation in Europe and North America [17–21], has been demonstrated for many organisms in Taiwan, e.g. plants, ground beetles, and stag beetles [12, 81–84]. The CMR is another major geographic barrier for genetic differentiation of extant organisms in populations of plants, fishes, frogs, toads, bats, crabs, and stag beetles [12, 35, 37, 85–88], subspecies of damselflies [34], and species of snails, fishes, tree frogs, lizards, crabs, crickets, and carabids [56, 83, 84, 89–94]. Hypotheses regarding the evolutionary history, under the hindrance of CMR and periodical glaciations during Pleistocene, for the montane stag beetles exhibiting morphological variations in this study were proposed.

Discordant relationships between morphological and molecular data have been found in several Formosan stag beetles. Huang and Lin [28] confirmed the three mandible forms in *L*. *formosanus* were related to the environment heterogeneity instead of genetic differentiation. Similar results were also observed in montane stag beetle *N*. *doro* because their characteristic elytra luster and coloration were significantly related to their habitat rather than genetic differentiation/subspecies [12].

Populations with unique morphological features caused by geographical isolation and recurrent glaciations are occasionally recognized as subspecies. Several subspecies isolated in different islands/regions were confirmed by molecular data in stag beetle *Dorcus titanus*, a species with 20 subspecies widely distributed in East and Southeast Asia [95]. Though examples of within-island subspeciation events are rare, they have been demonstrated in some cases in Taiwan [34, 38–40]. The results herein reveal each of the two geographic subspecies in *L*. *kanoi* and *L*. *ogakii* might have also been cases of within-island subspeciation. Although STRUCTURE analysis shows one cluster only for each of them, both PTP and *BEAST analyses find two geographic lineages for each of *L*. *ogakii* and *L*. *kanoi*, i.e. *L*. *ogakii* in eastern Taiwan with subspecies *L*. *o*. *ogakii* in the north and subspecies *L*. *o*. *chuyunshanus* in the south, and *L*. *kanoi* in western Taiwan with *L*. *k*. *piceus* in the north and *L*. *k*. *kanoi* in the center (Figs 1 and 5). Although the subspecific status of the two subspecies of *Pri*. *davidis*, i.e. *Pri*. *d*. *nigerrimus* and *Pri*. *d*. *cheni* are not completely supported by species delimitation, the phylogenetic monophyly, distinct genetic divergences in mtDNA/nuclear DNA, and the divergent time show it is reasonable to recognize their subspecific status, i.e. *Pri*. *d*. *nigerrimus* in the northern/eastern Taiwan and *Pri*. *d*. *cheni* in the midwest/southwest (Figs 1 and 6).

A more complicated evolutionary history has been illustrated in *N*. *swinhoei* complex: *N*. *swinhoei*, *N*. *eugeniae*, and *N*. *doro*, instead of being three species, are considered as a single taxon by Tsai et al. [12] due to their locally morphological variations and a genetic admixture resulting from the periodical glaciation events and mountain hindrance. A similar situation has also been found in montane leaf beetles which exhibited distinct morphological features and yet, have a genetic admixture [96]. Likewise, molecular evidences in this study show a complex differentiation history in montane lucanids of *L*. *ogakii*, *L*. *kanoi*, and *L*. *m*. *taiwanus*. Phylogenetic monophyly and species delimitation show *L*. *ogakii* and *L*. *kanoi* were isolated on each side of CMR (Figs 1 and 5). Hybridization might have occurred between morphologically distinct *L*. *m*. *taiwanus* and *L*. *k*. *kanoi*. The STRUCTURE analysis showed a possible introgression/hybridization event between them and the species delimitation by *BEAST and PTP also suggested *L*. *m*. *taiwanus* and *L*. *k*. *kanoi* are indistinguishable (Fig 5). Meanwhile, the ML tree conducted by the nuclear gene *wingless* also observed a genetic admixture of *L*. *ogakii*, *L*. *kanoi*, and *L*. *m*. *taiwanus* (S1 Fig). Since *L*. *m*. *taiwanus* is widespread throughout the entire island at altitudes ranging from 1,000–2,800 m, introgression/hybridization events might have occurred in these three closely related *Lucanus* stag beetles. Further studies including more samples and molecular markers are necessary to elucidate their complicated evolutionary history.

The calibration dating based on mitochondrial genes could help in clarifying the divergence time and providing additional information on the subspecific status of these montane stag beetles. It appears the ancestor of *L*. *ogakii* and *L*. *kanoi*, likely having arrived in Taiwan prior to 2.7 Mya in late Pliocene, was isolated in a drastic uplift event during 1–3 Mya on each side of the CMR (Figs 1 and 3A) [29, 30]. Subsequent geographic isolation ca. 1 Mya and thereafter local adaptation might have induced subspecific differentiation for both *L*. *ogakii* and *L*. *kanoi*. Afterwards, *L*. *maculifemoratus*, a species with several affinity subspecies recorded in Japan, Korea, and mainland China, dispersed to Taiwan prior to 0.68 Mya in the middle Pleistocene (Fig 3A). Introgression/hybridization events between *L*. *m*. *taiwanus* and *L*. *k*. *kanoi* shown in STRUCTURE analysis might have occurred, ca. 0.05–0.12 Mya in late Pleistocene (Figs 3A and 5). Moreover, the divergence time of the two subspecies of *Pri*. *davidis* could be traced back to ca. 0.7 Mya, i.e. middle Pleistocene (Fig 3B).

### Taxonomic delineation and genetic divergence

The nucleotide divergence distribution of mitochondrial COI gene, which is, >6% among most species, can be applied concordantly to species identification. Multilocus data examined in this study, such as the genetic divergence distribution of COI and 16S rDNA (Fig 2), could be used to discriminate most of the lucanid species. Somewhat lower divergence has been found in the more conservative 16S rDNA. Nevertheless, it would be difficult to make taxonomic recognition of a few genetically admixed species with either >2% intraspecies or <2% interspecies COI sequence variations. Indeed, Nunes et al. [50] pointed out the lack of a clear DNA boundary, such as a barcoding gap, might have resulted from recent genetic divergence, incomplete lineage sorting, and introgression [97–100]. Thus, more genetic markers including maternal mtDNA and biparental nuclear genes would be helpful to make further comprehensive taxonomic revision.

Out of 54 lucanid species and subspecies, two geographical subspecies have been recorded for each of the four montane stag beetles, i.e. *L*. *kanoi*, *L*. *ogakii*, *Pri*. *davidis*, and *N*. *doro*. Subspecies *L*. *k*. *piceus*, with nitidous and more inconspicuous hairs of elytra, could be distinguished from *L*. *k*. *kanoi*. On the basis of few differences in male/female genitalia, the difference of broad clypeus from *L*. *k*. *kanoi*, and the distinct frontal ridge of head, *L*. *ogakii* was downgraded to the third subspecies of *L*. *kanoi* [42]. In a recent revision, *Pri*. *d*. *nigerrimus* was treated as a synonym of *Pri*. *d*. *cheni* because the diagnostic characteristic, i.e. darker body color, used to distinguish them is overlapping [43]. Obviously, slightly different and variable morphological characters appear to be insufficient for delineating these mentioned subspecies.

Species boundary test and/or hybridization estimation for taxonomically debated taxa have been extensively applied in recent years [54, 101–108]. Though the individuals of the debated taxa were found genetically admixed in the same cluster by STRUCTURE analysis, PTP analyses shows the monophyly for the two subspecies of both *L*. *ogakii* and *L*. *kanoi* (Fig 5). The relatively high genetic divergence of COI and 16S rDNA is additional evidence for their subspecific status (Fig 3D). After examining a large number of samples and data collected on pertinent DNA markers, Tsai et al. [12] proposed *N*. *doro*, once considered to have two subspecies, should be regarded as a single taxon, and this is again supported by the STRUCTURE analysis herein (Fig 7). Considering the phylogenetic monophyly, genetic divergences in mtDNA/nuclear DNA, and divergence time, we believe *Pri*. *davidis* is composed of two geographic subspecies.

It is a difficult task for taxonomists to delineate closely related species, as demonstrated in *N*. *swinhoei* complex by Tsai et al. [12], when molecular evidences are incongruent with morphological characteristics among known species. Huang & Chen [43] considered the species status of *Pri*. *formosanus* and *Pri*. *piluensis* ambiguous because of the overlapping characteristics of the head/mandible and body coloration. In the present study, these two species have been shown to be indistinguishable because phylogenetic analyses revealed *Pri*. *piluensis* was admixed with *Pri*. *formosanus* and they were grouped as one single cluster by the STRUCTURE analysis (Figs 6 and 8). For the three montane *Lucanus* species, i.e. *L*. *ogakii*, *L*. *kanoi*, and *L*. *m*. *taiwanus*, molecular evidences show hybridization might have occurred (S1 Fig). *Lucanus m*. *taiwanus*, with a characteristically larger body size, curved level of mandible, and dorsal gold hair, can be clearly distinguished from the other two species, *L*. *ogakii* and *L*. *kanoi*, but the genetic distances of COI and 16S rDNA and relationships analyses show *L*. *k*. *kanoi* was genetically embedded in *L*. *m*. *taiwanus* (Figs 3A, 3D, 8A and 8B). The STRUCTURE analysis also suggested introgression/hybridization events might have occurred between *L*. *m*. *taiwanus* and *L*. *k*. *kanoi* (Fig 5).

Finally, this study has helped solve the taxonomical problem involving *D*. *mochizukii*, *D*. *formosanus*, *D*. *kyanrauensis*, and *D*. *parvulus*. These species lack typical *Dorcus* features and thus had been moved to genera *Falcicornis*, *Miwanus*, *Serrognathus*, and *Metallactulus* [44]. Phylogenetic inferences based on COI+16S rDNA+28S rDNA sequences herein suggest *Dorcus* is a suitable category for them (Fig 4).

## Supporting Information

S1 Fig Maximum likelihood (ML) trees based on the nuclear gene *wingless* for five *Lucanus* (A) and four *Prismognathus* (B) taxa are shown. The heterogeneous positions observed from the chromatogram are marked.
(TIFF)

S1 Table Information of Taxon ID, collection locality, GPS coordinates, and accession numbers of studied genes of each stag beetle. Sequences downloaded from GenBank for genetic divergence analysis are listed below the dashed line.
(DOC)

S2 Table Heterogeneous positions detected in *wingless* sequence chromatogram among *Lucanus kanoi*, *L*. *maculifemoratus taiwanus*, and *L*. *ogakii*. (DOC)

S3 Table Heterogeneous positions detected in *wingless* sequence chromatogram between *Pri*. *davidis cheni* and *Pri*. *d*. *nigerrimus* and that between *Pri*. *formosanus* and *Pri*. *piluensis*. (DOC)
