# Supplementary figures and images for: Subspecific Differentiation Events of Montane Stag Beetles (Coleoptera, Lucanidae) Endemic to Formosa Island
Source: PLoS One. 2016 Jun 3;11(6):e0156600. doi: 10.1371/journal.pone.0156600 (PMC4892689; doi:10.1371/journal.pone.0156600)

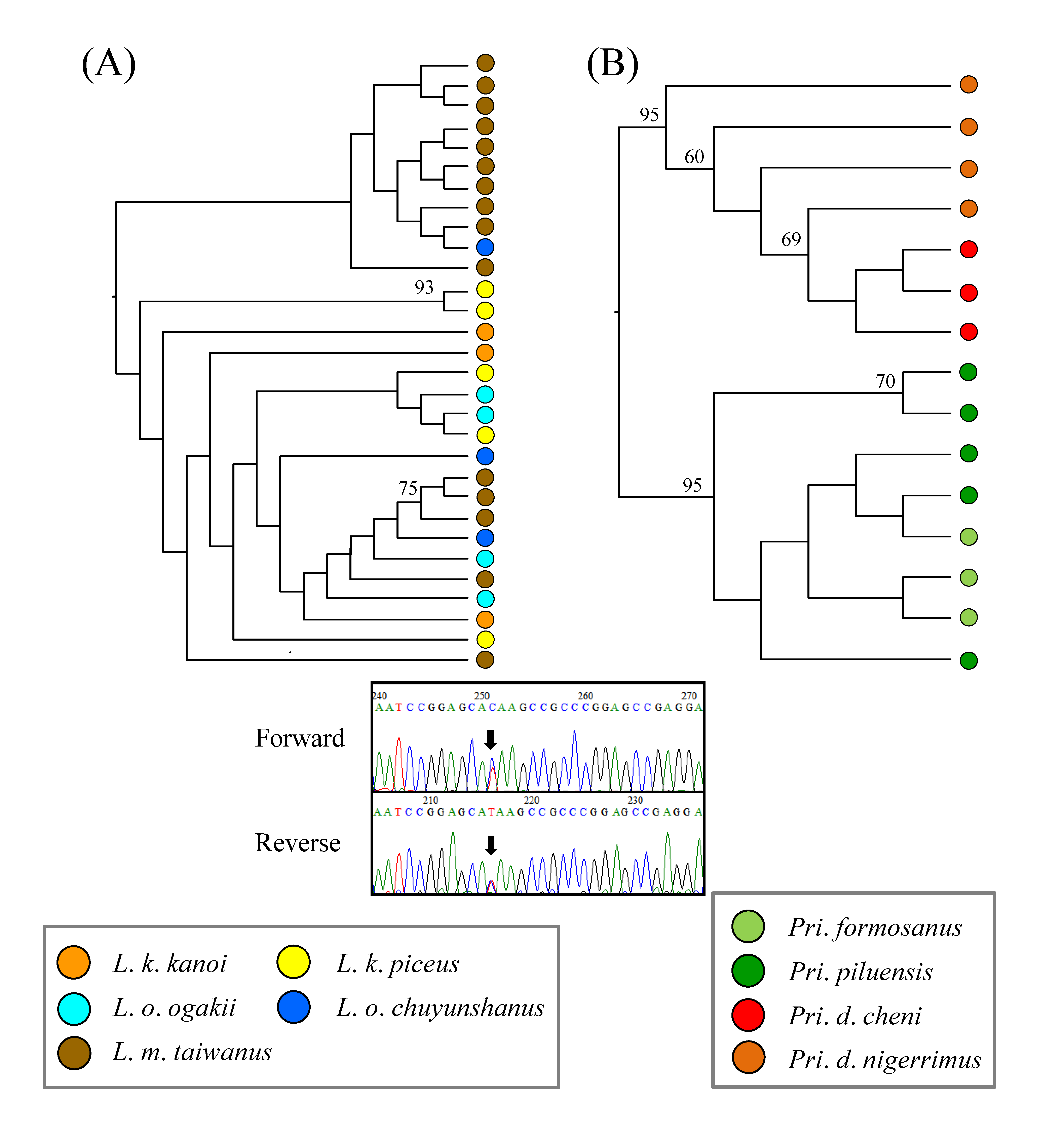

Supplement: S1 Fig — The heterogeneous positions observed from the chromatogram are marked. (TIFF) [file pone.0156600.s001.tiff]
