# Supplementary material for: Subspecific Differentiation Events of Montane Stag Beetles (Coleoptera, Lucanidae) Endemic to Formosa Island
Source: PLoS One. 2016 Jun 3;11(6):e0156600. doi: 10.1371/journal.pone.0156600 (PMC4892689; doi:10.1371/journal.pone.0156600)
Supplement: S1 Table — Sequences downloaded from GenBank for genetic divergence analysis are listed below the dashed line. (DOC) [file pone.0156600.s002.doc]

**S1 Table. Information of Taxon ID, collection locality, GPS coordinates, and accession numbers of studied genes of each stag beetle.** Sequences downloaded from GenBank for genetic divergence analysis are listed below the dashed line.

| **Taxon** | **ID** | | | **Collecting Locality** | | **GPS-latitude** | | **GPS-longitude** | | **COI** | | **16S rDNA** | | **28S rDNA** | | ***wingless*** | |
| --- | --- | --- | --- | --- | --- | --- | --- | --- | --- | --- | --- | --- | --- | --- | --- | --- | --- |
| *Dorcus carinulatus* | Luc143 | | | Zhonghe, Taichung | | 24.160805° | | 120.840670° | | LC074471 | | LC074974 | | LC066683 | | *-* | |
| *Dorcus carinulatus* | Luc144 | | | Zhonghe, Taichung | | 24.160805° | | 120.840670° | | LC074472 | | LC074975 | | LC066684 | | *-* | |
| *Dorcus carinulatus* | Luc711 | | | Zhonghe, Taichung | | 24.160805° | | 120.840670° | | LC074473 | | LC074976 | | LC066685 | | *-* | |
| *Dorcus gracilicornis* | Luc471 | | | Anma Mountain, Taichung | | 24.241647° | | 120.944452° | | LC074474 | | LC074977 | | LC066686 | | *-* | |
| *Dorcus gracilicornis* | Luc659 | | | Anma Mountain, Taichung | | 24.227004° | | 120.974000° | | LC074475 | | LC074978 | | LC066687 | | *-* | |
| *Dorcus gracilicornis* | Luc675 | | | Anma Mountain, Taichung | | 24.227004° | | 120.974000° | | LC074476 | | LC074979 | | LC066688 | | *-* | |
| *Dorcus kyanrauensis* | Luc019 | | | Lalashan, Taoyuan | | 24.690047° | | 121.409929° | | LC074477 | | - | | - | | *-* | |
| *Dorcus kyanrauensis* | Luc226 | | | Luhu, Miaoli | | 24.540353° | | 121.037075° | | LC074478 | | LC074980 | | LC066689 | | *-* | |
| *Dorcus kyanrauensis* | Luc243 | | | Baguali, Miaoli | | 24.531664° | | 120.962857° | | LC074479 | | LC074981 | | LC066690 | | *-* | |
| *Dorcus kyanrauensis* | Luc408 | | | Luhu, Miaoli | | 24.540834° | | 121.027512° | | LC074480 | | LC074982 | | LC066691 | | *-* | |
| *Dorcus miwai* | Luc027 | | | Shanlinxi, Nantou | | 23.655291° | | 120.783183° | | LC074481 | | - | | - | | *-* | |
| *Dorcus miwai* | Luc029 | | | Shanlinxi, Nantou | | 23.655291° | | 120.783183° | | LC074482 | | LC074983 | | LC066692 | | *-* | |
| *Dorcus miwai* | Luc080 | | | Wulin, Taichung | | 24.363670° | | 121.302699° | | LC074483 | | LC074984 | | LC066693 | | *-* | |
| *Dorcus miwai* | Luc156 | | | Lalashan, Taoyuan | | 24.702151° | | 121.419716° | | LC074484 | | LC074985 | | LC066694 | | *-* | |
| *Dorcus miwai* | Luc163 | | | Bilu, Hualien | | 24.180132° | | 121.403901° | | LC074485 | | LC074986 | | LC066695 | | *-* | |
| *Dorcus miwai* | Luc561 | | | Anma Mountain, Taichung | | 24.241569° | | 120.945517° | | LC074486 | | LC074987 | | LC066696 | | *-* | |
| *Dorcus mochizukii* | Luc275 | | | Luchang, Miaoli | | 24.540834° | | 121.027512° | | LC074487 | | LC074988 | | LC066697 | | *-* | |
| *Dorcus mochizukii* | Luc665 | | | Dinghu, Chiayi | | 23.490225° | | 120.718700° | | LC074488 | | LC074989 | | LC066698 | | *-* | |
| *Dorcus mochizukii* | Luc1091 | | | Siyuan, Taichung | | 24.397240° | | 121.362535° | | LC074489 | | LC074990 | | LC066699 | | *-* | |
| *Dorcus parvulus* | Luc729 | | | Lanyu, Taitung | | 22.028442° | | 121.556981° | | LC074490 | | LC074991 | | LC066700 | | *-* | |
| *Dorcus parvulus* | Luc743 | | | Lanyu, Taitung | | 22.028442° | | 121.556981° | | LC074491 | | LC074992 | | LC066701 | | *-* | |
| *Dorcus parvulus* | Luc744 | | | Lanyu, Taitung | | 22.028442° | | 121.556981° | | LC074492 | | LC074993 | | LC066702 | | *-* | |
| *Dorcus reichei clypeatus* | Luc052 | | | Northern Cross-Island Highway, Taoyuan | | 24.702151° | | 121.419716° | | LC074493 | | LC074994 | | LC066703 | | *-* | |
| *Dorcus reichei clypeatus* | Luc305 | | | Syuejian, Miaoli | | 24.409468° | | 121.005077° | | LC074494 | | LC074995 | | LC066704 | | *-* | |
| *Dorcus reichei clypeatus* | Luc306 | | | Syuejian, Miaoli | | 24.409468° | | 121.005077° | | LC074495 | | LC074996 | | LC066705 | | *-* | |
| *Dorcus reichei clypeatus* | Luc315 | | | Shanlinxi, Nantou | | 23.655434° | | 120.777880° | | LC074496 | | LC074997 | | LC066706 | | *-* | |
| *Dorcus reichei clypeatus* | Luc316 | | | Shanlinxi, Nantou | | 23.655434° | | 120.777880° | | LC074497 | | LC074998 | | LC066707 | | *-* | |
| *Dorcus reichei clypeatus* | Luc524 | | | Jianshi,Hsinchu | | 24.680855° | | 121.294765° | | LC074498 | | LC074999 | | LC066708 | | *-* | |
| *Dorcus reichei clypeatus* | Luc553 | | | Dadong Mountain, Chiayi | | 23.493211° | | 120.721915° | | LC074499 | | LC075000 | | LC066709 | | *-* | |
| *Dorcus striatipennis yushiroi* | Luc229 | | | Anma Mountain, Taichung | | 24.233793° | | 120.981578° | | LC074500 | | LC075001 | | LC066710 | | *-* | |
| *Dorcus striatipennis yushiroi* | Luc287 | | | Anma Mountain, Taichung | | 24.233793° | | 120.981578° | | LC074501 | | LC075002 | | LC066711 | | *-* | |
| *Dorcus striatipennis yushiroi* | Luc694 | | | Anma Mountain, Taichung | | 24.241569° | | 120.945517° | | LC074502 | | LC075003 | | LC066712 | | *-* | |
| **Taxon** | **ID** | **Collecting Locality** | | | **GPS-latitude** | | **GPS-longitude** | | **COI** | | **16S rDNA** | | **28S rDNA** | | ***wingless*** | |  |
| *Dorcus taiwanicus* | Luc002 | National Chung Hsing University, Taichung | | | 24.120689° | | 120.674942° | | LC074503 | | - | | - | | *-* | |  |
| *Dorcus taiwanicus* | Luc031 | Shanlinxi, Nantou | | | 23.655291° | | 120.783183° | | LC074504 | | LC075004 | | LC066713 | | *-* | |  |
| *Dorcus taiwanicus* | Luc037 | Nanshanxi, Nantou | | | 24.014895° | | 121.090836° | | LC074505 | | LC075005 | | LC066714 | | *-* | |  |
| *Dorcus taiwanicus* | Luc046 | Sanmin, Kaohsiung | | | - | | - | | LC074506 | | LC075006 | | LC066715 | | *-* | |  |
| *Dorcus taiwanicus* | Luc053 | Nanshanxi, Nantou | | | 24.014895° | | 121.090836° | | LC074507 | | LC075007 | | LC066716 | | *-* | |  |
| *Dorcus titanus sika* | Luc217 | Luchang, Miaoli | | | 24.540834° | | 121.027512° | | LC074508 | | LC075008 | | LC066717 | | *-* | |  |
| *Dorcus titanus sika* | Luc227 | Xitou, Nantou | | | 23.709836° | | 120.777090° | | LC074509 | | LC075009 | | LC066718 | | *-* | |  |
| *Dorcus titanus sika* | Luc320 | Beipu, Hsinchu | | | 24.671287° | | 121.056600° | | LC074510 | | LC075010 | | LC066719 | | *-* | |  |
| *Dorcus yamadai* | Luc271 | Meifeng, Nantou | | | 24.093505° | | 121.176322° | | LC074511 | | LC075011 | | LC066720 | | *-* | |  |
| *Dorcus yamadai* | Luc645 | Guanyuan, Hualien | | | 24.734000° | | 121.116611° | | LC074512 | | LC075012 | | LC066721 | | *-* | |  |
| *Dorcus yamadai* | Luc684 | Siyuan, Taichung | | | 24.397098° | | 121.356497° | | LC074513 | | LC075013 | | LC066722 | | *-* | |  |
| *Nigidius acutangulus* | Luc522 | Taian, Miaoli | | | 24.470706° | | 120.977927° | | LC074514 | | LC075014 | | LC066723 | | *-* | |  |
| *Nigidius acutangulus* | Luc565 | Basian Mountain, Taichung | | | 24.192774° | | 121.017122° | | LC074515 | | LC075015 | | LC066724 | | *-* | |  |
| *Nigidius acutangulus* | Luc567 | Huisun Forest Area, Nantou | | | 24.091139° | | 121.041028° | | LC074516 | | LC075016 | | LC066725 | | *-* | |  |
| *Nigidius baeri* | Luc162 | Lanyu, Taitung | | | 22.028442° | | 121.556981° | | LC074517 | | LC075017 | | LC066726 | | *-* | |  |
| *Nigidius baeri* | Luc165 | Lanyu, Taitung | | | 22.028442° | | 121.556981° | | LC074518 | | LC075018 | | LC066728 | | *-* | |  |
| *Nigidius baeri* | Luc572 | Lanyu, Taitung | | | 22.028442° | | 121.556981° | | LC074519 | | LC075019 | | LC066727 | | *-* | |  |
| *Nigidius formosanus* | Luc145 | Dakeng, Taichung | | | 24.173968° | | 120.781924° | | - | | LC075020 | | - | | *-* | |  |
| *Nigidius formosanus* | Luc463 | Lilong Mountain, Pingtung | | | 22.158166° | | 120.724774° | | LC074520 | | LC075021 | | LC066729 | | *-* | |  |
| *Nigidius formosanus* | Luc529 | Kenting, Pingtung | | | 21.959537° | | 120.802011° | | LC074521 | | LC075022 | | LC066730 | | *-* | |  |
| *Nigidius lewisi* | Luc1086 | Shouka, Pingtung | | | 22.252546° | | 120.838858° | | LC074522 | | LC075023 | | LC066731 | | *-* | |  |
| *Nigidius lewisi* | Luc1087 | Shouka, Pingtung | | | 22.252546° | | 120.838858° | | LC074523 | | LC075024 | | LC066732 | | *-* | |  |
| *Nigidius lewisi* | Luc1088 | Shouka, Pingtung | | | 22.252546° | | 120.838858° | | LC074524 | | LC075025 | | LC066733 | | *-* | |  |
| *Aegus chelifer* | Luc710 | Madou, Tainan | | | 22.683333° | | 120.533333° | | LC074525 | | LC075026 | | LC066734 | | *-* | |  |
| *Aegus chelifer* | Luc776 | Pingtung | | | - | | - | | LC074526 | | LC075027 | | LC066735 | | *-* | |  |
| *Aegus chelifer* | Luc777 | Pingtung | | | - | | - | | LC074527 | | LC075028 | | LC066736 | | *-* | |  |
| *Aegus chelifer* | Luc779 | Madou, Tainan | | | - | | - | | LC074528 | | LC075029 | | LC066737 | | *-* | |  |
| *Aegus kurosawai* | Luc482 | Anma Mountain, Taichung | | | 24.224624° | | 120.973308° | | LC074529 | | LC075030 | | LC066738 | | *-* | |  |
| *Aegus kurosawai* | Luc1093 | Beidongyan Mountain, Nantou | | | 24.070339° | | 121.129204° | | LC074530 | | LC075031 | | LC066739 | | *-* | |  |
| *Aegus kurosawai* | Luc1094 | Beidongyan Mountain, Nantou | | | 24.070339° | | 121.129204° | | LC074531 | | LC075032 | | LC066740 | | *-* | |  |
| *Aegus laevicollis formosae* | Luc208 | Shanlinxi, Nantou | | | 23.653324° | | 120.781244° | | LC074532 | | LC075034 | | LC066741 | | *-* | |  |
| *Aegus laevicollis formosae* | Luc272 | Meifeng, Nantou | | | 24.093505° | | 121.176322° | | LC074533 | | LC075035 | | LC066742 | | *-* | |  |
| *Aegus laevicollis formosae* | Luc497 | Luchang, Miaoli | | | 24.540834° | | 121.027512° | | LC074534 | | LC075036 | | LC066743 | | *-* | |  |
| *Aegus laevicollis formosae* | Luc129 | Taman Mountain, Taoyuan | | | 24.698954° | | 121.427462° | | - | | LC075033 | | LC066744 | | *-* | |  |
| *Aegus nakaneorum* | Luc121 | Tengzhi, Kaohsiung | | | 23.054261° | | 120.720881° | | LC074535 | | - | | - | | *-* | |  |
| **Taxon** | **ID** | | **Collecting Locality** | | **GPS-latitude** | | **GPS-longitude** | | **COI** | | **16S rDNA** | | **28S rDNA** | | ***wingless*** | | |
| *Aegus nakaneorum* | Luc517 | | Gukuan, Taichung | | 24.171178° | | 120.944759° | | LC074536 | | LC075037 | | LC066745 | | *-* | | |
| *Aegus nakaneorum* | Luc519 | | Gukuan, Taichung | | 24.171178° | | 120.944759° | | AB762278 | | AB762649 | | LC066746 | | *-* | | |
| *Aegus nakaneorum* | Luc568 | | Huisun Forest Area, Nantou | | 24.087740° | | 121.034149° | | - | | - | | LC066747 | | *-* | | |
| *Prismognathus davidis cheni* | Luc554 | | Meifeng, Nantou | | 24.065035° | | 121.166044° | | LC074537 | | LC075038 | | LC066748 | | LC126097 | | |
| *Prismognathus davidis cheni* | Luc1290 | | Alishan, Chiayi | | - | | - | | LC074538 | | LC075039 | | LC066749 | | LC126092 | | |
| *Prismognathus davidis cheni* | Luc1291 | | Alishan, Chiayi | | - | | - | | - | | LC075040 | | LC066750 | | *-* | | |
| *Prismognathus davidis cheni* | Luc1292 | | Alishan, Chiayi | | - | | - | | LC074539 | | LC075041 | | LC066751 | | LC126093 | | |
| *Prismognathus davidis nigerrimus* | Luc245 | | Bilu, Hualien | | 24.183319° | | 121.386035° | | LC074540 | | LC075042 | | LC066752 | | LC126098 | | |
| *Prismognathus davidis nigerrimus* | Luc726 | | Bilu, Hualien | | 24.183319° | | 121.386035° | | LC074541 | | LC075043 | | LC066753 | | LC126096 | | |
| *Prismognathus davidis nigerrimus* | Luc727 | | Bilu, Hualien | | 24.183319° | | 121.386035° | | LC074542 | | LC075044 | | LC066754 | | LC126090 | | |
| *Prismognathus davidis nigerrimus* | Luc1066 | | Syuejian, Miaoli | | 24.424611° | | 121.013363° | | LC074543 | | LC075045 | | LC066755 | | LC126091 | | |
| *Prismognathus formosanus* | Luc102 | | Songgang, Nantou | | 24.062695° | | 121.165252° | | LC074544 | | LC075046 | | LC066756 | | LC126089 | | |
| *Prismognathus formosanus* | Luc728 | | Xiangyang, Taitung | | 23.246013° | | 120.993166° | | LC074545 | | LC075047 | | LC066757 | | LC126088 | | |
| *Prismognathus formosanus* | Luc1073 | | Songgang, Nantou | | 24.062695° | | 121.165252° | | LC074546 | | LC075048 | | LC066758 | | LC126087 | | |
| *Prismognathus piluensis* | Luc472 | | Anma Mountain, Taichung | | 24.224624° | | 120.973308° | | LC074547 | | LC075049 | | LC066759 | | LC126084 | | |
| *Prismognathus piluensis* | Luc558 | | Anma Mountain, Taichung | | 24.224624° | | 120.973308° | | LC074548 | | LC075050 | | LC066760 | | LC126086 | | |
| *Prismognathus piluensis* | Luc559 | | Anma Mountain, Taichung | | 24.224624° | | 120.973308° | | LC074549 | | LC075051 | | LC066761 | | LC126085 | | |
| *Prismognathus piluensis* | Luc1297 | | Guanyuan, Hualien | | 24.185547° | | 121.325906° | | LC091038 | | LC091040 | | LC126100 | | LC126094 | | |
| *Prismognathus piluensis* | Luc1298 | | Guanyuan, Hualien | | 24.185547° | | 121.325906° | | LC091039 | | LC091041 | | LC126101 | | LC126095 | | |
| *Lucanus datunensis* | Luc131 | | Datun Mountain, Taipei | | 25.174038° | | 121.522101° | | LC074550 | | LC075052 | | LC066762 | | *-* | | |
| *Lucanus datunensis* | Luc1056 | | Datun Mountain, Taipei | | 25.174038° | | 121.522101° | | LC074551 | | LC075053 | | LC066763 | | *-* | | |
| *Lucanus datunensis* | Luc1092 | | Datun Mountain, Taipei | | 25.174038° | | 121.522101° | | LC074552 | | LC075054 | | LC066764 | | *-* | | |
| *Lucanus formosanus* | Luc008 | | Cingjing, Nantou | | 24.043116° | | 121.147097 | | LC074553 | | LC075055 | | LC066765 | | *-* | | |
| *Lucanus formosanus* | Luc009 | | Lugu, Nantou | | 23.679519° | | 120.792080° | | LC074554 | | LC075056 | | LC066766 | | *-* | | |
| *Lucanus formosanus* | Luc113 | | Chunyang, Nantou | | 24.028412° | | 121.148001° | | AB762280 | | AB762650 | | LC066767 | | *-* | | |
| *Lucanus formosanus* | Luc114 | | Chunyang, Nantou | | 24.028412° | | 121.148001° | | LC074555 | | LC075057 | | LC066768 | | *-* | | |
| *Lucanus kanoi kanoi* | Luc544 | | Songgang, Nantou | | 24.065035° | | 121.166044° | | LC074556 | | LC075058 | | LC066769 | | LC077663 | | |
| *Lucanus kanoi kanoi* | Luc545 | | Songgang, Nantou | | 24.065035° | | 121.166044° | | LC074557 | | LC075059 | | LC066770 | | LC077664 | | |
| *Lucanus kanoi kanoi* | Luc555 | | Songgang, Nantou | | 24.065035° | | 121.166044° | | LC074558 | | LC075060 | | LC066771 | | LC077665 | | |
| *Lucanus kanoi piceus* | Luc741 | | Siyuan, Taichung | | 24.397240° | | 121.362535° | | LC074559 | | LC075061 | | LC066772 | | LC077666 | | |
| *Lucanus kanoi piceus* | Luc1049 | | Siyuan, Taichung | | 24.397240° | | 121.362535° | | LC074560 | | - | | - | | - | | |
| *Lucanus kanoi piceus* | Luc1095 | | Siyuan, Taichung | | 24.397240° | | 121.362535° | | LC074561 | | LC075063 | | LC066774 | | LC077667 | | |
| *Lucanus kanoi piceus* | Luc1096 | | Siyuan, Taichung | | 24.397240° | | 121.362535° | | LC074562 | | LC075064 | | LC066775 | | LC077668 | | |
| *Lucanus kanoi piceus* | Luc1097 | | Siyuan, Taichung | | 24.397240° | | 121.362535° | | LC074563 | | LC075065 | | LC066776 | | LC077669 | | |
| *Lucanus kanoi piceus* | Luc1098 | | Siyuan, Taichung | | 24.397240° | | 121.362535° | | LC074564 | | LC075066 | | LC066777 | | LC077670 | | |
| **Taxon** | **ID** | | **Collecting Locality** | | **GPS-latitude** | | **GPS-longitude** | | **COI** | | **16S rDNA** | | **28S rDNA** | | ***wingless*** | | |
| *Lucanus kurosawai* | Luc525 | | Alishan, Chiayi | | 23.497656° | | 120.818533° | | LC074565 | | LC075067 | | LC066778 | | - | | |
| *Lucanus kurosawai* | Luc546 | | Anma Mountain, Taichung | | 24.261834° | | 120.994646 | | LC074566 | | LC075068 | | LC066779 | | LC126099 | | |
| *Lucanus kurosawai* | Luc547 | | Anma Mountain, Taichung | | 24.261834° | | 120.994646 | | LC074567 | | LC075069 | | LC066780 | | - | | |
| *Lucanus kurosawai* | Luc548 | | Anma Mountain, Taichung | | 24.261834° | | 120.994646 | | LC074568 | | LC075070 | | LC066781 | | - | | |
| *Lucanus maculifemoratus taiwanus* | Luc112 | | Tengzhi, Kaohsiung | | 23.066452° | | 120.751821° | | - | | LC075071 | | LC066783 | | LC077671 | | |
| *Lucanus maculifemoratus taiwanus* | Luc179 | | Xiangyang, Taitung | | 23.246013° | | 120.993166° | | - | | - | | LC066784 | | - | | |
| *Lucanus maculifemoratus taiwanus* | Luc188 | | Anma Mountain, Taichung | | 24.254845° | | 121.008781° | | LC074569 | | LC075072 | | LC066782 | | LC077672 | | |
| *Lucanus maculifemoratus taiwanus* | Luc189 | | Anma Mountain, Taichung | | 24.254845° | | 121.008781° | | LC074570 | | LC075073 | | LC066785 | | LC077673 | | |
| *Lucanus maculifemoratus taiwanus* | Luc193 | | Cingjing, Nantou | | 24.065035° | | 121.166044° | | LC074571 | | LC075074 | | LC066786 | | LC077674 | | |
| *Lucanus maculifemoratus taiwanus* | Luc194 | | Cingjing, Nantou | | 24.065035° | | 121.166044° | | LC074572 | | LC075075 | | LC066787 | | LC077675 | | |
| *Lucanus maculifemoratus taiwanus* | Luc197 | | Cingjing, Nantou | | 24.065035° | | 121.166044° | | LC074573 | | LC075076 | | LC066788 | | LC077676 | | |
| *Lucanus maculifemoratus taiwanus* | Luc198 | | Cingjing, Nantou | | 24.065035° | | 121.166044° | | LC074574 | | LC075077 | | LC066789 | | LC077677 | | |
| *Lucanus maculifemoratus taiwanus* | Luc207 | | Wulin, Taichung | | 24.363670° | | 121.302699° | | LC074575 | | LC075078 | | LC066790 | | LC077678 | | |
| *Lucanus maculifemoratus taiwanus* | Luc211 | | Luchang, Miaoli | | 24.540834° | | 121.027512° | | - | | LC075079 | | LC066791 | | - | | |
| *Lucanus maculifemoratus taiwanus* | Luc498 | | Anma Mountain, Taichung | | 24.271713° | | 120.985582° | | LC074576 | | LC075080 | | LC066792 | | LC077679 | | |
| *Lucanus maculifemoratus taiwanus* | Luc499 | | Anma Mountain, Taichung | | 24.271713° | | 120.985582° | | LC074577 | | LC075081 | | LC066793 | | LC077680 | | |
| *Lucanus maculifemoratus taiwanus* | Luc500 | | Anma Mountain, Taichung | | 24.271713° | | 120.985582° | | LC074578 | | LC075082 | | LC066794 | | LC077681 | | |
| *Lucanus maculifemoratus taiwanus* | Luc501 | | Anma Mountain, Taichung | | 24.271713° | | 120.985582° | | LC074579 | | LC075083 | | LC066795 | | LC077682 | | |
| *Lucanus maculifemoratus taiwanus* | Luc502 | | Anma Mountain, Taichung | | 24.271713° | | 120.985582° | | LC074580 | | LC075084 | | LC066796 | | LC077683 | | |
| *Lucanus maculifemoratus taiwanus* | Luc562 | | Wulin, Taichung | | 24.363670° | | 121.302699° | | LC074581 | | LC075085 | | LC066797 | | LC077684 | | |
| *Lucanus maculifemoratus taiwanus* | Luc787 | | Wulin, Taichung | | 24.363670° | | 121.302699° | | LC074582 | | LC075062 | | LC066773 | | LC077685 | | |
| *Lucanus maculifemoratus taiwanus* | Luc1102 | | Xinbaiyang, Hualien | | 24.197804° | | 121.432729° | | LC074583 | | LC075086 | | LC066798 | | LC077686 | | |
| *Lucanus miwai* | Luc167 | | Songgang, Nantou | | 24.065035° | | 121.166044° | | LC074584 | | LC075087 | | LC066799 | | - | | |
| *Lucanus miwai* | Luc503 | | Jianshi, Hsinchu | | 24.668809° | | 121.281465° | | LC074585 | | LC075088 | | LC066800 | | - | | |
| *Lucanus miwai* | Luc526 | | Songgang, Nantou | | 24.065035° | | 121.166044° | | LC074586 | | LC075089 | | LC066801 | | - | | |
| *Lucanus miwai* | Luc527 | | Songgang, Nantou | | 24.065035° | | 121.166044° | | LC074587 | | LC075090 | | LC066802 | | - | | |
| *Lucanus miwai* | Luc528 | | Songgang, Nantou | | 24.065035° | | 121.166044° | | LC074588 | | LC075091 | | LC066803 | | - | | |
| *Lucanus ogakii chuyunshanus* | Luc132 | | Xiangyang, Taitung | | 23.246013° | | 120.993166° | | LC074589 | | LC075092 | | - | | - | | |
| *Lucanus ogakii chuyunshanus* | Luc542 | | Xiangyang, Taitung | | 23.246013° | | 120.993166° | | LC074590 | | LC075093 | | LC066804 | | LC077687 | | |
| *Lucanus ogakii chuyunshanus* | Luc543 | | Xiangyang, Taitung | | 23.246013° | | 120.993166° | | LC074591 | | LC075094 | | LC066805 | | LC077688 | | |
| *Lucanus ogakii chuyunshanus* | Luc1101 | | Xiangyang, Taitung | | 23.246013° | | 120.993166° | | LC074592 | | LC075095 | | LC066806 | | LC077689 | | |
| *Lucanus ogakii ogakii* | Luc111 | | Bilu, Hualien | | 24.180132° | | 121.403901° | | LC074593 | | LC075096 | | LC066807 | | LC077690 | | |
| *Lucanus ogakii ogakii* | Luc135 | | Bilu, Hualien | | 24.180132° | | 121.403901° | | LC074594 | | LC075097 | | LC066808 | | LC077691 | | |
| *Lucanus ogakii ogakii* | Luc200 | | Bilu, Hualien | | 24.180132° | | 121.403901° | | LC074595 | | LC075098 | | LC066809 | | LC077692 | | |
| *Lucanus ogakii ogakii* | Luc1100 | | Guanyuan, Hualien | | 24.181094° | | 121.344188° | | LC074596 | | LC075099 | | LC066810 | | LC077693 | | |
| **Taxon** | **ID** | | **Collecting Locality** | | **GPS-latitude** | | **GPS-longitude** | | **COI** | | **16S rDNA** | | **28S rDNA** | | ***wingless*** | | |
| *Lucanus swinhoei* | Luc191 | | Songgang, Nantou | | 24.065035° | | 121.166044° | | LC074597 | | LC075100 | | LC066811 | | - | | |
| *Lucanus swinhoei* | Luc509 | | Northern Cross-Island Highway, Taoyuan | | 24.655369° | | 121.399704° | | LC074598 | | LC075101 | | LC066812 | | - | | |
| *Lucanus swinhoei* | Luc742 | | Guangfu, Hualien | | 23.668181° | | 121.373435° | | LC074599 | | LC075102 | | LC066813 | | - | | |
| *Cyclommatus asahinai* | Luc232 | | Luchang, Miaoli | | 24.540834° | | 121.027512° | | LC074600 | | LC075103 | | LC066814 | | - | | |
| *Cyclommatus asahinai* | Luc233 | | Luchang, Miaoli | | 24.540834° | | 121.027512° | | LC074601 | | LC075104 | | LC066815 | | - | | |
| *Cyclommatus asahinai* | Luc335 | | Anma Mountain, Taichung | | 24.229201° | | 120.979150° | | LC074602 | | LC075105 | | LC066816 | | - | | |
| *Cyclommatus mniszechi* | Luc651 | | Sanxia, Taipei | | 24.831994° | | 121.445449° | | LC074603 | | LC075106 | | LC066817 | | - | | |
| *Cyclommatus mniszechi* | Luc1279 | | Taipei | | - | | - | | LC074604 | | LC075107 | | LC066818 | | - | | |
| *Cyclommatus mniszechi* | Luc1293 | | Sanxia, Taipei | | 24.889846° | | 121.426694° | | LC074605 | | LC075108 | | LC066819 | | - | | |
| *Cyclommatus scutellaris* | Luc234 | | Luhu, Miaoli | | 24.540834° | | 121.027512° | | LC074606 | | LC075109 | | LC066820 | | - | | |
| *Cyclommatus scutellaris* | Luc235 | | Luhu, Miaoli | | 24.540834° | | 121.027512° | | LC074607 | | LC075110 | | LC066821 | | - | | |
| *Cyclommatus scutellaris* | Luc237 | | Luhu, Miaoli | | 24.540834° | | 121.027512° | | LC074608 | | LC075111 | | LC066822 | | - | | |
| *Cyclommatus scutellaris* | Luc270 | | Xitou, Nantou | | 23.711934° | | 120.779403° | | LC074609 | | LC075112 | | LC066823 | | - | | |
| *Rhaetulus crenatus crenatus* | Luc011 | | Baling, Taoyuan | | 24.680238° | | 121.379866° | | LC074610 | | LC075113 | | LC066824 | | - | | |
| *Rhaetulus crenatus crenatus* | Luc097 | | Wushe, Nantou | | 24.022873° | | 121.131771° | | LC074611 | | LC075114 | | LC066825 | | - | | |
| *Rhaetulus crenatus crenatus* | Luc118 | | Bihu, Nantou | | 24.023484° | | 121.133311° | | LC074612 | | LC075115 | | LC066826 | | - | | |
| *Aesalus imanishii* | Luc1289 | | Anma Mountain, Taichung | | 24.224624° | | 120.973308° | | LC074613 | | LC075116 | | LC066827 | | - | | |
| *Pseudorhaetus sinicus concolor* | Luc164 | | Dahan Mountain, Pingtung | | - | | - | | LC074614 | | - | | - | | - | | |
| *Pseudorhaetus sinicus concolor* | Luc239 | | Luchang, Miaoli | | 24.540834° | | 121.027512° | | LC074615 | | LC075117 | | LC066828 | | - | | |
| *Pseudorhaetus sinicus concolor* | Luc577 | | Luchang, Miaoli | | 24.540834° | | 121.027512° | | LC074616 | | LC075118 | | LC066829 | | - | | |
| *Pseudorhaetus sinicus concolor* | Luc626 | | Xinbaiyang, Hualien | | 24.205078° | | 121.444351° | | LC074617 | | LC075119 | | LC066830 | | - | | |
| *Neolucanus doro doro* | Luc183(AM01) | | Anma Mountain, Taichung City | | 24.227823° | | 120.970330° | | AB762102 | | AB762476 | | - | | AB765424 | | |
| *Neolucanus doro doro* | Luc185(AM02) | | Anma Mountain, Taichung City | | 24.227823° | | 120.970330° | | AB762103 | | AB762477 | | - | | AB765425 | | |
| *Neolucanus doro doro* | Luc186(AM03) | | Anma Mountain, Taichung City | | 24.227823° | | 120.970330° | | AB762104 | | AB762478 | | - | | AB765426 | | |
| *Neolucanus doro doro* | Luc276(AM04) | | Anma Mountain, Taichung City | | 24.227823° | | 120.970330° | | AB762105 | | AB762479 | | - | | AB765427 | | |
| *Neolucanus doro doro* | Luc277(AM05) | | Anma Mountain, Taichung City | | 24.227823° | | 120.970330° | | AB762106 | | AB762480 | | - | | AB765428 | | |
| *Neolucanus doro doro* | Luc578(HW03) | | Hewang Mountain, Nantou | | 24.11805° | | 121.186687° | | AB762141 | | AB762515 | | LC066831 | | AB765462 | | |
| *Neolucanus doro doro* | Luc580(HW05) | | Hewang Mountain, Nantou | | 24.11805° | | 121.186687° | | AB762143 | | AB762517 | | LC066832 | | AB765464 | | |
| *Neolucanus doro doro* | Luc585(HW10) | | Hewang Mountain, Nantou | | 24.11805° | | 121.186687° | | AB762148 | | AB762522 | | - | | AB765469 | | |
| *Neolucanus doro doro* | Luc596(HW12) | | Hewang Mountain, Nantou | | 24.11805° | | 121.186687° | | AB762150 | | AB762524 | | LC066833 | | AB765471 | | |
| *Neolucanus doro doro* | Luc598(HW14) | | Hewang Mountain, Nantou | | 24.11805° | | 121.186687° | | AB762152 | | AB762526 | | - | | AB765473 | | |
| *Neolucanus doro horaguchii* | Luc417(GW02) | | Guanwu, Hsinchu | | 24.525874° | | 121.114176° | | AB762125 | | AB762499 | | LC066834 | | AB765446 | | |
| *Neolucanus doro horaguchii* | Luc437(GW10) | | Guanwu, Hsinchu | | 24.525874° | | 121.114176° | | AB762133 | | AB762507 | | LC066835 | | AB765454 | | |
| *Neolucanus doro horaguchii* | Luc438(GW11) | | Guanwu, Hsinchu | | 24.525874° | | 121.114176° | | AB762134 | | AB762508 | | LC066836 | | AB765455 | | |
| *Neolucanus doro horaguchii* | Luc439(GW12) | | Guanwu, Hsinchu | | 24.525874° | | 121.114176° | | AB762135 | | AB762509 | | LC066837 | | AB765456 | | |
| **Taxon** | **ID** | | **Collecting Locality** | | **GPS-latitude** | | **GPS-longitude** | | **COI** | | **16S rDNA** | | **28S rDNA** | | ***wingless*** | | |
| *Neolucanus doro horaguchii* | LucAF021(GW13) | | Guanwu, Hsinchu | | 24.525874° | | 121.114176° | | AB762136 | | AB762510 | | - | | AB765457 | | |
| *Neolucanus doro horaguchii* | Luc304(SJ01) | | Syuejian, Miaoli | | 24.409468° | | 121.005077° | | AB762156 | | AB762530 | | - | | AB765477 | | |
| *Neolucanus doro horaguchii* | Luc312(SJ02) | | Syuejian, Miaoli | | 24.409468° | | 121.005077° | | AB762157 | | AB762531 | | - | | AB765478 | | |
| *Neolucanus doro horaguchii* | Luc313(SJ03) | | Syuejian, Miaoli | | 24.409468° | | 121.005077° | | AB762158 | | AB762532 | | - | | AB765479 | | |
| *Neolucanus doro horaguchii* | Luc351(SJ04) | | Syuejian, Miaoli | | 24.409468° | | 121.005077° | | AB762159 | | AB762533 | | - | | AB765480 | | |
| *Neolucanus doro horaguchii* | Luc352(SJ05) | | Syuejian, Miaoli | | 24.409468° | | 121.005077° | | AB762160 | | AB762534 | | - | | AB765481 | | |
| *Neolucanus eugeniae* | LucAF116(SP01) | | Shanping, Kaohsiung | | 22.992011° | | 120.690278° | | AB762211 | | AB762585 | | LC066838 | | AB765530 | | |
| *Neolucanus eugeniae* | LucAF117(SP02) | | Shanping, Kaohsiung | | 22.992011° | | 120.690278° | | AB762212 | | AB762586 | | LC066839 | | AB765531 | | |
| *Neolucanus eugeniae* | LucAF118(SP03) | | Shanping, Kaohsiung | | 22.992011° | | 120.690278° | | AB762213 | | AB762587 | | LC066840 | | AB765532 | | |
| *Neolucanus eugeniae* | LucAF120(SP04) | | Shanping, Kaohsiung | | 22.992011° | | 120.690278° | | AB762214 | | AB762588 | | - | | AB765533 | | |
| *Neolucanus eugeniae* | LucAF125(SP06) | | Shanping, Kaohsiung | | 22.992011° | | 120.690278° | | AB762216 | | AB762589 | | - | | AB765535 | | |
| *Neolucanus eugeniae* | LucAF127(SP08) | | Shanping, Kaohsiung | | 22.992011° | | 120.690278° | | AB762218 | | AB762590 | | - | | AB765537 | | |
| *Neolucanus maximus vendli* | Luc484 | | Shanlinxi, Nantou | | 23.653324° | | 120.781244° | | AB762275 | | AB762646 | | LC066841 | | - | | |
| *Neolucanus maximus vendli* | Luc485 | | Shanlinxi, Nantou | | 23.653324° | | 120.781244° | | AB762276 | | AB762647 | | LC066842 | | - | | |
| *Neolucanus maximus vendli* | Luc692 | | Jianshi, Hsinchu | | 24.680855° | | 121.294765° | | LC074618 | | LC075120 | | LC066843 | | - | | |
| *Neolucanus sinicus taiwanus* | Luc535 | | Pinglin, Taipei | | 24.971923° | | 121.770727° | | LC074619 | | LC075121 | | LC066844 | | - | | |
| *Neolucanus sinicus taiwanus* | Luc537 | | Pinglin, Taipei | | 24.971923° | | 121.770727° | | LC074620 | | LC075122 | | LC066845 | | - | | |
| *Neolucanus sinicus taiwanus* | Luc539 | | Pinglin, Taipei | | 24.971923° | | 121.770727° | | LC074621 | | LC075123 | | LC066846 | | - | | |
| *Neolucanus sinicus* | LucAF123 | | Jianshi, Hsinchu | | 24.676587° | | 121.315316° | | LC074622 | | - | | - | | - | | |
| *Neolucanus swinhoei* | Luc106(TP02) | | Yingge, Taipei | | 24.985206° | | 121.353294° | | AB762222 | | AB762602 | | LC066847 | | AB765548 | | |
| *Neolucanus swinhoei* | Luc380(ALS10) | | Alishan, Chiayi | | 23.484882° | | 120.717942° | | AB762225 | | AB762605 | | LC066848 | | AB765550 | | |
| *Neolucanus swinhoei* | Luc381(ALS11) | | Alishan, Chiayi | | 23.484882° | | 120.717942° | | AB762226 | | AB762606 | | LC066849 | | AB765551 | | |
| *Neolucanus swinhoei* | Luc390(ALS14) | | Alishan, Chiayi | | 23.490634° | | 120.753384° | | AB762229 | | AB762609 | | LC066850 | | AB765553 | | |
| *Neolucanus swinhoei* | Luc429(ML05) | | Dakeng, Taichung | | 24.169186° | | 120.799796° | | AB762235 | | AB762615 | | LC066851 | | AB765559 | | |
| *Neolucanus swinhoei* | Luc443(AM19) | | Anma Mountain, Taichung | | 24.247364° | | 120.913782° | | AB762236 | | AB762616 | | LC066852 | | AB765560 | | |
| *Neolucanus swinhoei* | Luc444(AM20) | | Anma Mountain, Taichung | | 24.248344° | | 120.918482° | | AB762237 | | AB762617 | | LC066853 | | AB765561 | | |
| *Neolucanus swinhoei* | Luc445(AM21) | | Anma Mountain, Taichung | | 24.246901° | | 120.936170° | | AB762238 | | AB762618 | | LC066854 | | AB765562 | | |
| *Neolucanus swinhoei* | Luc446(AM22) | | Anma Mountain, Taichung | | 24.241802° | | 120.943710° | | AB762239 | | AB762619 | | LC066855 | | AB765563 | | |
| *Neolucanus swinhoei* | Luc487(ML07) | | Sanyi, Miaoli | | 24.380857° | | 120.788036° | | AB762244 | | AB762624 | | LC066856 | | AB765568 | | |
| *Neolucanus swinhoei* | Luc488(ML08) | | Sanyi, Miaoli | | 24.380857° | | 120.788036° | | AB762245 | | AB762625 | | LC066857 | | AB765569 | | |
| *Neolucanus swinhoei* | Luc595(ML11) | | Mingchi, Ilan | | 24.649402 | | 121.423068 | | AB762248 | | AB762628 | | LC066858 | | AB765571 | | |
| *Neolucanus swinhoei* | Luc605(SP11) | | Shanping, Kaohsiung | | 22.970365° | | 120.684973° | | AB762251 | | AB762631 | | LC066859 | | AB765574 | | |
| *Neolucanus swinhoei* | Luc606(SP12) | | Shanping, Kaohsiung | | 22.970365° | | 120.684973° | | AB762252 | | AB762632 | | LC066860 | | AB765575 | | |
| *Neolucanus swinhoei* | Luc607(SP13) | | Shanping, Kaohsiung | | 22.970365° | | 120.684973° | | AB762253 | | AB762633 | | LC066861 | | AB765576 | | |
| *Neolucanus swinhoei* | Luc618(SP14) | | Tengzhi, Kaohsiung | | 23.017584° | | 120.693312° | | AB762254 | | AB762634 | | LC066862 | | AB765577 | | |
| **Taxon** | **ID** | | **Collecting Locality** | | **GPS-latitude** | | **GPS-longitude** | | **COI** | | **16S rDNA** | | **28S rDNA** | | ***wingless*** | | |
| *Neolucanus swinhoei* | Luc621(TP04) | | Shiding, Taipei | | 24.683833° | | 121.620417° | | AB762255 | | AB762635 | | LC066863 | | AB765578 | | |
| *Neolucanus swinhoei* | Luc622(TP05) | | Shiding, Taipei | | 24.683833° | | 121.620417° | | AB762256 | | AB762636 | | LC066864 | | AB765579 | | |
| *Neolucanus swinhoei* | Luc623(TP06) | | Shiding, Taipei | | 24.683833° | | 121.620417° | | AB762257 | | AB762637 | | LC066865 | | AB765580 | | |
| *Neolucanus swinhoei* | Luc624(TP07) | | Shiding, Taipei | | 24.683833° | | 121.620417° | | AB762258 | | AB762638 | | LC066866 | | AB765581 | | |
| *Neolucanus swinhoei* | Luc625(TP08) | | Shiding, Taipei | | 24.683833° | | 121.620417° | | AB762259 | | AB762639 | | LC066867 | | AB765582 | | |
| *Neolucanus swinhoei* | Luc685(TP09) | | Shulin, Taipei | | 25.050080° | | 121.403447° | | AB762260 | | AB762640 | | LC066868 | | AB765583 | | |
| *Neolucanus swinhoei* | Luc686(TP10) | | Shulin, Taipei | | 25.050080° | | 121.403447° | | AB762261 | | AB762641 | | LC066869 | | AB765584 | | |
| *Neolucanus swinhoei* | Luc687(TP11) | | Shulin, Taipei | | 25.050080° | | 121.403447° | | AB762262 | | AB762642 | | LC066870 | | AB765585 | | |
| *Neolucanus swinhoei* | Luc713 | | Ruisui, Hualien | | 23.504742° | | 121.304151° | | LC074623 | | LC075124 | | LC066871 | | - | | |
| *Neolucanus swinhoei* | Luc714 | | Ruisui, Hualien | | 23.504742° | | 121.304151° | | LC074624 | | LC075125 | | LC066872 | | - | | |
| *Neolucanus swinhoei* | Luc715 | | Ruisui, Hualien | | 23.504742° | | 121.304151° | | LC074625 | | LC075126 | | LC066873 | | - | | |
| *Neolucanus swinhoei* | Luc717 | | Tongmen, Hualien | | 24.008193° | | 121.395469° | | LC074626 | | LC075127 | | LC066874 | | - | | |
| *Neolucanus swinhoei* | Luc718 | | Tongmen, Hualien | | 24.008193° | | 121.395469° | | LC074627 | | LC075128 | | LC066875 | | - | | |
| *Neolucanus swinhoei* | Luc740 | | Dahan Mountain, Pingtung | | 22.413289° | | 120.752743° | | LC074628 | | LC075129 | | LC066876 | | - | | |
| *Neolucanus swinhoei* | Luc804 | | Taman Mountain, Taoyuan | | 24.698373° | | 121.422129° | | LC074629 | | LC075130 | | LC066877 | | - | | |
| *Neolucanus swinhoei* | Luc808 | | Taman Mountain, Taoyuan | | 24.698373° | | 121.422129° | | LC074630 | | LC075131 | | LC066878 | | - | | |
| *Neolucanus swinhoei* | Luc809 | | Taman Mountain, Taoyuan | | 24.698373° | | 121.422129° | | LC074631 | | LC075132 | | LC066879 | | - | | |
| *Neolucanus swinhoei* | Luc951 | | Daoxia, Hsinchu | | 24.669913° | | 121.264780° | | LC074632 | | LC075133 | | LC066880 | | - | | |
| *Neolucanus swinhoei* | Luc952 | | Daoxia, Hsinchu | | 24.669913° | | 121.264780° | | LC074633 | | LC075134 | | LC066881 | | - | | |
| *Neolucanus swinhoei* | Luc957 | | Yulao, Hsinchu | | 24.664208 | | 121.277522 | | LC074634 | | LC075135 | | LC066882 | | - | | |
| *Neolucanus swinhoei* | Luc963 | | Mingchi, Ilan | | 24.648867° | | 121.466209° | | LC074635 | | LC075136 | | LC066883 | | - | | |
| *Neolucanus swinhoei* | Luc964 | | Mingchi, Ilan | | 24.648867° | | 121.466209° | | LC074636 | | LC075137 | | LC066884 | | - | | |
| *Neolucanus swinhoei* | Luc1039 | | Provincial highway No. 9, Ilan | | 24.868758° | | 121.772013° | | LC074637 | | LC075138 | | LC066885 | | - | | |
| *Neolucanus swinhoei* | Luc1045 | | Ruisui, Hualien | | 23.504742° | | 121.304151° | | LC074638 | | LC075139 | | LC066886 | | - | | |
| *Neolucanus swinhoei* | Luc1046 | | Jean-Zen Mountain, Taitung | | 22.627804° | | 120.954337° | | LC074639 | | LC075140 | | LC066887 | | - | | |
| *Neolucanus swinhoei* | Luc1047 | | Jean-Zen Mountain, Taitung | | 22.627804° | | 120.954337° | | LC074640 | | LC075141 | | LC066888 | | - | | |
| *Neolucanus swinhoei* | Luc1078 | | Leshui, Ilan | | 24.595437° | | 121.531668° | | LC074641 | | LC075142 | | LC066889 | | - | | |
| *Neolucanus swinhoei* | Luc1079 | | Leshui, Ilan | | 24.595437° | | 121.531668° | | LC074642 | | LC075143 | | LC066890 | | - | | |
| *Neolucanus swinhoei* | Luc1126 | | Taiping Mountain, Ilan | | 24.534549° | | 121.519133° | | LC074643 | | LC075144 | | LC066891 | | - | | |
| *Neolucanus swinhoei* | Luc1127 | | Taiping Mountain, Ilan | | 24.534549° | | 121.519133° | | LC074644 | | LC075145 | | LC066892 | | - | | |
| *Neolucanus swinhoei* | Luc1163 | | Dahan Mountain, Pingtung | | 22.408538° | | 120.756146° | | LC074645 | | LC075146 | | LC066893 | | - | | |
| *Neolucanus swinhoei* | Luc1177 | | Dahan Mountain, Pingtung | | 22.408538° | | 120.756146° | | LC074646 | | LC075147 | | LC066894 | | - | | |
| *Neolucanus swinhoei* | Luc1232 | | Liushishi Mountain, Hualien | | 23.224365° | | 121.316732° | | LC074647 | | LC075148 | | LC066895 | | - | | |
| *Neolucanus swinhoei* | Luc1233 | | Liushishi Mountain, Hualien | | 23.224365° | | 121.316732° | | LC074648 | | LC075149 | | LC066896 | | - | | |
| *Neolucanus swinhoei* | Luc1234 | | Chike Mountain, Hualien | | 23.388087° | | 121.391670° | | LC074649 | | LC075150 | | LC066897 | | - | | |
| **Taxon** | **ID** | | **Collecting Locality** | | **GPS-latitude** | | **GPS-longitude** | | **COI** | | **16S rDNA** | | **28S rDNA** | | ***wingless*** | | |
| *Neolucanus swinhoei* | Luc1235 | | Chike Mountain, Hualien | | 23.388087° | | 121.391670° | | LC074650 | | LC075151 | | LC066898 | | - | | |
| *Neolucanus swinhoei* | Luc1236 | | Chike Mountain, Hualien | | 23.388087° | | 121.391670° | | LC074651 | | LC075152 | | LC066899 | | - | | |
| *Neolucanus swinhoei* | Luc1237 | | Chike Mountain, Hualien | | 23.388087° | | 121.391670° | | LC074652 | | LC075153 | | LC066900 | | - | | |
| *Figulus binodulus* | Luc505 | | Dakeng, Taichung | | 24.173968° | | 120.7819° | | LC074653 | | LC075154 | | LC066901 | | - | | |
| *Figulus binodulus* | Luc506 | | Dakeng, Taichung | | 24.173968° | | 120.7819° | | LC074654 | | LC075155 | | LC066902 | | - | | |
| *Figulus binodulus* | Luc512 | | Dakeng, Taichung | | 24.173968° | | 120.7819° | | LC074655 | | LC075156 | | LC066903 | | - | | |
| *Figulus punctatus* | Luc518 | | Changle, Pingtung | | 22.079599° | | 120.841465° | | LC074656 | | LC075157 | | LC066904 | | - | | |
| *Figulus punctatus* | Luc1069 | | Shouka, Pingtung | | 22.252546° | | 120.838858° | | LC074657 | | LC075158 | | LC066905 | | - | | |
| *Figulus punctatus* | Luc1070 | | Shouka, Pingtung | | 22.252546° | | 120.838858° | | LC074658 | | LC075159 | | LC066906 | | - | | |
| *Nigidionus parryi* | Luc078 | | Basian Mountain, Taichung | | 24.195584° | | 121.011484° | | LC074659 | | LC075160 | | LC066907 | | - | | |
| *Nigidionus parryi* | Luc184 | | Anma Mountain, Taichung | | 24.242238° | | 120.909684° | | LC074660 | | LC075161 | | LC066908 | | - | | |
| *Nigidionus parryi* | Luc336 | | Anma Mountain, Taichung | | 24.244364° | | 120.934498° | | LC074661 | | LC075162 | | LC066909 | | - | | |
| *Nigidionus parryi* | Luc422 | | Tannan, Nantou | | 23.823315° | | 120.939524° | | LC074662 | | LC075163 | | LC066910 | | - | | |
| *Nigidionus parryi* | Luc514 | | Anma Mountain, Taichung | | 24.244364° | | 120.934498° | | LC074663 | | LC075164 | | LC066911 | | - | | |
| *Prosopocoilus astacoides blanchardi* | Luc017 | | Baling, Taoyuan | | 24.680238° | | 121.379866° | | LC074664 | | LC075165 | | LC066912 | | - | | |
| *Prosopocoilus astacoides blanchardi* | Luc038 | | Jianshi, Hsinchu | | - | | - | | LC074665 | | LC075166 | | LC066913 | | - | | |
| *Prosopocoilus astacoides blanchardi* | Luc057 | | Xibao, Hualien | | 24.191242° | | 121.480988° | | LC074666 | | LC075167 | | LC066914 | | - | | |
| *Prosopocoilus astacoides blanchardi* | Luc126 | | Taman Mountain, Taoyuan | | 24.698373° | | 121.422129° | | LC074667 | | - | | - | | - | | |
| *Prosopocoilus astacoides blanchardi* | Luc138 | | Lichia, Taitung | | 22.807318° | | 121.029897° | | LC074668 | | LC075168 | | LC066915 | | - | | |
| *Prosopocoilus astacoides blanchardi* | Luc139 | | Lichia, Taitung | | 22.807318° | | 121.029897° | | LC074669 | | LC075169 | | LC066916 | | - | | |
| *Prosopocoilus astacoides blanchardi* | Luc236 | | Luchang, Miaoli | | 121.027512° | | 24.540834° | | LC074670 | | LC075170 | | LC066917 | | - | | |
| *Prosopocoilus forficula austerus* | Luc109 | | Shinhsin, Taoyuan | | 24.637446° | | 121.402868° | | LC074671 | | LC075171 | | LC066918 | | - | | |
| *Prosopocoilus forficula austerus* | Luc691 | | Aowanda, Nantou | | 23.957565° | | 121.160359° | | LC074672 | | LC075172 | | LC066919 | | - | | |
| *Dorcus formosanus* | Luc107 | | Shanlinxi, Nantou | | 120.781244° | | 23.653324° | | LC074673 | | LC075173 | | LC066920 | | - | | |
| *Dorcus formosanus* | Luc213 | | Luchang, Miaoli | | 121.027512° | | 24.540834° | | LC074674 | | LC075174 | | LC066921 | | - | | |
| *Dorcus formosanus* | Luc214 | | Luchang, Miaoli | | 121.027512° | | 24.540834° | | LC074675 | | LC075175 | | LC066922 | | - | | |
| *Dorcus formosanus* | Luc339 | | Shanlinxi, Nantou | | 120.781244° | | 23.653324° | | LC074676 | | LC075176 | | LC066923 | | - | | |
| *Prosopocoilus motschulskii* | Luc141 | | Xihu, Changhua | | - | | - | | LC074677 | | LC075177 | | LC066924 | | - | | |
| *Prosopocoilus motschulskii* | Luc520 | | Hemei, Changhua | | - | | - | | LC074678 | | LC075178 | | LC066925 | | - | | |
| *Prosopocoilus motschulskii* | Luc1076 | | Houli, Taichung | | 24.298033° | | 120.736326° | | LC074679 | | LC075179 | | LC066926 | | - | | |
| *Odontolabis siva parryi* | Luc024 | | Nanan, Hualien | | - | | - | | LC074680 | | - | | - | | - | | |
| *Odontolabis siva parryi* | Luc033 | | Gukuan, Taichung | | 24.171178° | | 120.944759° | | LC074681 | | LC075181 | | LC066927 | | - | | |
| *Odontolabis siva parryi* | Luc108 | | Gukuan, Taichung | | 24.171178° | | 120.944759° | | LC074682 | | - | | - | | - | | |
| *Odontolabis siva parryi* | Luc218 | | Luchang, Miaoli | | 24.540834° | | 121.027512° | | LC074683 | | LC075180 | | LC066928 | | - | | |
| *Odontolabis siva parryi* | Luc360 | | Syuejian, Miaoli | | 24.401712° | | 120.945211° | | AB762279 | | AB762651 | | LC066929 | | - | | |
| **Taxon** | **ID** | | **Collecting Locality** | | **GPS-latitude** | | **GPS-longitude** | | **COI** | | **16S rDNA** | | **28S rDNA** | | ***wingless*** | | |
| *Odontolabis siva parryi* | Luc421 | | Bailan, Hsinchu | | 24.581733° | | 121.091321° | | LC074684 | | LC075182 | | LC066930 | | *-* | | |
| *Xylotrupes mniszechi tonkinensis* | Scar01 | | Green Island, Taitung | | 22.637647° | | 121.498810° | | LC074690 | | LC075188 | | LC066936 | | *-* | | |
| *Allomyrina dichotoma tunobosonis* | Scar02 | | Chiayi | | 23.361307° | | 120.581643° | | LC074686 | | LC075184 | | LC066932 | | *-* | | |
| *Anomala aulacoides* | Scar03 | | Anma Mountain, Taichung | | 24.248388° | | 120.918155° | | LC074687 | | LC075185 | | LC066933 | | *-* | | |
| *Leptaulax formosanus* | Pas02 | | Syuejian, Miaoli | | 24.405425° | | 120.995594° | | LC074688 | | LC075186 | | LC066934 | | *-* | | |
| *Aceraius grandis* | Pas03 | | Syuejian, Miaoli | | 24.405425° | | 120.995594° | | LC074685 | | LC075183 | | LC066931 | | *-* | | |
| *Phelotrupes formosanus* | Geo01 | | Xue Mountain, Taichung | | 24.384428° | | 121.294350° | | LC074689 | | LC075187 | | LC066935 | | *-* | | |
| *Ceruchus sp.* | - | | Canada: Ontario, Point Pelee, Point Pelee National Park | | - | | - | | GU013657 | | - | | - | | *-* | | |
| *Ceruchus chrysomelinus* | - | | Germany: Bavaria, Bayerischer Wald | | - | | - | | KM445089 | | - | | - | | *-* | | |
| *Ceruchus chrysomelinus* | - | | Germany: Bavaria, Bayerischer Wald | | - | | - | | KM443704 | | - | | - | | *-* | | |
| *Ceruchus chrysomelinus* | - | | Germany: Bavaria, Bayerischer Wald | | - | | - | | KM443701 | | - | | - | | *-* | | |
| *Ceruchus chrysomelinus* | - | | Germany: Bavaria, Bayerischer Wald | | - | | - | | KM439280 | | - | | - | | *-* | | |
| *Dorcus parallelipipedus* | - | | Sweden: Moensteras, Stroemsrum | | - | | - | | KJ964713 | | - | | - | | *-* | | |
| *Dorcus parallelipipedus* | - | | Sweden: Moerbylanga, Stora Roer | | - | | - | | KJ965240 | | - | | - | | *-* | | |
| *Dorcus parallelipipedus* | - | | Germany: Rhineland Palatinate, Noerdliche Oberrheinebene | | - | | - | | KM442856 | | - | | - | | *-* | | |
| *Dorcus parallelipipedus* | - | | Germany: North Rhine-Westphalia, Niederrheinische Bucht | | - | | - | | KM452034 | | - | | - | | *-* | | |
| *Dorcus curvidens binodulosus* | - | | Japan:Yamanashi | | - | | - | | AB110716 | | - | | - | | *-* | | |
| *Dorcus curvidens binodulosus* | - | | Japan:Yamanashi | | - | | - | | AB110717 | | - | | - | | *-* | | |
| *Dorcus titanus okinawanus* | - | | Japan:Okinawa, Kunigami | | - | | - | | AB110718 | | - | | - | | *-* | | |
| *Dorcus titanus sika* | - | | Taiwan:Kaohsiung, Paolai | | - | | - | | AB110719 | | - | | - | | *-* | | |
| *Dorcus rectus rectus* | - | | Japan:Aomori, Hirosaki | | - | | - | | AB110720 | | - | | - | | *-* | | |
| *Dorcus rectus rectus* | - | | Japan:Aomori, Nishimeya, Meya dam | | - | | - | | AB110721 | | - | | - | | *-* | | |
| *Dorcus striatipennis striatipennis* | - | | Japan:Aomori, Towadako, Tsutanuma woodland pass | | - | | - | | AB110722 | | - | | - | | *-* | | |
| *Dorcus striatipennis striatipennis* | - | | Japan:Aomori, Iwaki, Mt. Iwaki | | - | | - | | AB110723 | | - | | - | | *-* | | |
| *Dorcus rubrofemoratus* | - | | Japan:Aomori, Nishimeya, Meya dam | | - | | - | | AB110724 | | - | | - | | *-* | | |
| *Dorcus rubrofemoratus* | - | | Japan:Aomori, Nishimeya | | - | | - | | AB110725 | | - | | - | | *-* | | |
| *Dorcus montivagus montivagus* | - | | Japan:Aomori, Kuroishi, Aonizawa | | - | | - | | AB110726 | | - | | - | | *-* | | |
| *Dorcus montivagus montivagus* | - | | Japan:Aomori, Ikarigaseki, Hisayoshi | | - | | - | | AB110727 | | - | | - | | *-* | | |
| *Lucanus formosanus* | - | | Taiwan | | - | | - | | FJ606582 | | - | | - | | *-* | | |
| *Lucanus formosanus* | - | | Taiwan | | - | | - | | FJ606583 | | - | | - | | *-* | | |
| *Lucanus formosanus* | - | | Taiwan | | - | | - | | FJ606584 | | - | | - | | *-* | | |
| *Lucanus formosanus* | - | | Taiwan | | - | | - | | FJ606585 | | - | | - | | *-* | | |
| *Lucanus formosanus* | - | | Taiwan | | - | | - | | FJ606586 | | - | | - | | *-* | | |
| *Lucanus formosanus* | - | | Taiwan | | - | | - | | FJ606587 | | - | | - | | *-* | | |
| *Lucanus formosanus* | - | | Taiwan | | - | | - | | FJ606588 | | - | | - | | *-* | | |
| **Taxon** | **ID** | | **Collecting Locality** | | **GPS-latitude** | | **GPS-longitude** | | **COI** | | **16S rDNA** | | **28S rDNA** | | ***wingless*** | | |
| *Lucanus formosanus* | - | | Taiwan | | - | | - | | FJ606589 | | - | | - | | *-* | | |
| *Lucanus formosanus* | - | | Taiwan | | - | | - | | FJ606590 | | - | | - | | *-* | | |
| *Lucanus formosanus* | - | | Taiwan | | - | | - | | FJ606591 | | - | | - | | *-* | | |
| *Lucanus formosanus* | - | | Taiwan | | - | | - | | FJ606592 | | - | | - | | *-* | | |
| *Lucanus formosanus* | - | | Taiwan | | - | | - | | FJ606593 | | - | | - | | *-* | | |
| *Lucanus formosanus* | - | | Taiwan | | - | | - | | FJ606594 | | - | | - | | *-* | | |
| *Lucanus formosanus* | - | | Taiwan | | - | | - | | FJ606595 | | - | | - | | *-* | | |
| *Lucanus formosanus* | - | | Taiwan | | - | | - | | FJ606596 | | - | | - | | *-* | | |
| *Lucanus formosanus* | - | | Taiwan | | - | | - | | FJ606597 | | - | | - | | *-* | | |
| *Lucanus formosanus* | - | | Taiwan | | - | | - | | FJ606598 | | - | | - | | *-* | | |
| *Lucanus formosanus* | - | | Taiwan | | - | | - | | FJ606599 | | - | | - | | *-* | | |
| *Lucanus formosanus* | - | | Taiwan | | - | | - | | FJ606600 | | - | | - | | *-* | | |
| *Lucanus formosanus* | - | | Taiwan | | - | | - | | FJ606601 | | - | | - | | *-* | | |
| *Lucanus formosanus* | - | | Taiwan | | - | | - | | FJ606602 | | - | | - | | *-* | | |
| *Lucanus formosanus* | - | | Taiwan | | - | | - | | FJ606603 | | - | | - | | *-* | | |
| *Lucanus formosanus* | - | | Taiwan | | - | | - | | FJ606604 | | - | | - | | *-* | | |
| *Lucanus formosanus* | - | | Taiwan | | - | | - | | FJ606605 | | - | | - | | *-* | | |
| *Lucanus formosanus* | - | | Taiwan | | - | | - | | FJ606606 | | - | | - | | *-* | | |
| *Lucanus formosanus* | - | | Taiwan | | - | | - | | FJ606607 | | - | | - | | *-* | | |
| *Lucanus formosanus* | - | | Taiwan | | - | | - | | FJ606608 | | - | | - | | *-* | | |
| *Lucanus formosanus* | - | | Taiwan | | - | | - | | FJ606609 | | - | | - | | *-* | | |
| *Lucanus formosanus* | - | | Taiwan | | - | | - | | FJ606610 | | - | | - | | *-* | | |
| *Lucanus formosanus* | - | | Taiwan | | - | | - | | FJ606611 | | - | | - | | *-* | | |
| *Lucanus formosanus* | - | | Taiwan | | - | | - | | FJ606612 | | - | | - | | *-* | | |
| *Lucanus formosanus* | - | | Taiwan | | - | | - | | FJ606613 | | - | | - | | *-* | | |
| *Lucanus formosanus* | - | | Taiwan | | - | | - | | FJ606614 | | - | | - | | *-* | | |
| *Lucanus formosanus* | - | | Taiwan | | - | | - | | FJ606615 | | - | | - | | *-* | | |
| *Lucanus formosanus* | - | | Taiwan | | - | | - | | FJ606616 | | - | | - | | *-* | | |
| *Lucanus formosanus* | - | | Taiwan | | - | | - | | FJ606617 | | - | | - | | *-* | | |
| *Lucanus formosanus* | - | | Taiwan | | - | | - | | FJ606618 | | - | | - | | *-* | | |
| *Lucanus formosanus* | - | | Taiwan | | - | | - | | FJ606619 | | - | | - | | *-* | | |
| *Lucanus formosanus* | - | | Taiwan | | - | | - | | FJ606620 | | - | | - | | *-* | | |
| *Lucanus formosanus* | - | | Taiwan | | - | | - | | FJ606621 | | - | | - | | *-* | | |
| *Lucanus formosanus* | - | | Taiwan | | - | | - | | FJ606622 | | - | | - | | *-* | | |
| *Lucanus formosanus* | - | | Taiwan | | - | | - | | FJ606623 | | - | | - | | *-* | | |
| **Taxon** | **ID** | | **Collecting Locality** | | **GPS-latitude** | | **GPS-longitude** | | **COI** | | **16S rDNA** | | **28S rDNA** | | ***wingless*** | | |
| *Lucanus formosanus* | **-** | | Taiwan | | - | | - | | FJ606624 | | **-** | | **-** | | ***-*** | | |
| *Lucanus formosanus* | **-** | | Taiwan | | - | | - | | FJ606625 | | **-** | | **-** | | ***-*** | | |
| *Lucanus formosanus* | **-** | | Taiwan | | - | | - | | FJ606626 | | **-** | | **-** | | ***-*** | | |
| *Lucanus formosanus* | **-** | | Taiwan | | - | | - | | FJ606627 | | **-** | | **-** | | ***-*** | | |
| *Lucanus formosanus* | **-** | | Taiwan | | - | | - | | FJ606628 | | **-** | | **-** | | ***-*** | | |
| *Lucanus formosanus* | **-** | | Taiwan | | - | | - | | FJ606629 | | **-** | | **-** | | ***-*** | | |
| *Lucanus formosanus* | **-** | | Taiwan | | - | | - | | FJ606630 | | **-** | | **-** | | ***-*** | | |
| *Lucanus formosanus* | **-** | | Taiwan | | - | | - | | FJ606631 | | **-** | | **-** | | ***-*** | | |
| *Lucanus formosanus* | **-** | | Taiwan | | - | | - | | FJ606632 | | **-** | | **-** | | ***-*** | | |
| *Lucanus formosanus* | **-** | | Taiwan | | - | | - | | FJ606633 | | **-** | | **-** | | ***-*** | | |
| *Lucanus fryi* | **-** | | Thailand:Chiang Mai | | - | | - | | FJ606539 | | **-** | | **-** | | ***-*** | | |
| *Lucanus laminifer laminifer* | **-** | | Thailand:Wiang Papao | | - | | - | | FJ606540 | | **-** | | **-** | | ***-*** | | |
| *Lucanus maculifemoratus taiwanus* | **-** | | Taiwan:Chiayi, Alishan | | - | | - | | FJ606541 | | **-** | | **-** | | ***-*** | | |
| *Lucanus maculifemoratus maculifemoratus* | **-** | | Japan:Aomori, Nishimeya, Meya dam | | - | | - | | AB110733 | | **-** | | **-** | | ***-*** | | |
| *Lucanus maculifemoratus maculifemoratus* | **-** | | Japan:Aomori, Nishimeya, Meya dam | | - | | - | | AB110734 | | **-** | | **-** | | ***-*** | | |
| *Lucanus sericeus* | **-** | | Thailand:Chiang Mai | | - | | - | | FJ606542 | | **-** | | **-** | | ***-*** | | |
| *Lucanus thibetanus isakii* | **-** | | Myanmar:Kachin | | - | | - | | FJ606543 | | **-** | | **-** | | ***-*** | | |
| *Lucanus ogakii* | **-** | | Taiwan:Taitung, Shiang-Yang | | - | | - | | FJ606544 | | **-** | | **-** | | ***-*** | | |
| *Lucanus datunensis* | **-** | | Taiwan:Taipei, Datunshan | | - | | - | | FJ606545 | | **-** | | **-** | | ***-*** | | |
| *Lucanus miwai* | **-** | | Taiwan:Nantou, Songkang | | - | | - | | FJ606546 | | **-** | | **-** | | ***-*** | | |
| *Lucanus swinhoei* | **-** | | Taiwan:Nantou, Songkang | | - | | - | | FJ606547 | | **-** | | **-** | | ***-*** | | |
| *Lucanus mazama* | **-** | | Unknown | | - | | - | | FJ613419 | | **-** | | **-** | | ***-*** | | |
| *Lucanus mazama* | **-** | | Unknown | | - | | - | | NC_013578 | | **-** | | **-** | | ***-*** | | |
| *Lucanus hayashii* | **-** | | Myanmar | | - | | - | | FJ606560 | | **-** | | **-** | | ***-*** | | |
| *Lucanus szetschuanicus szetschuanicus* | **-** | | China:Sichuan, Leshan | | - | | - | | FJ606561 | | **-** | | **-** | | ***-*** | | |
| *Lucanus hermani* | **-** | | China:Fujian, Wu Yi Shan | | - | | - | | FJ606552 | | **-** | | **-** | | ***-*** | | |
| *Lucanus planeti* | **-** | | China:Yunnan | | - | | - | | FJ606553 | | **-** | | **-** | | ***-*** | | |
| *Lucanus laetus* | **-** | | China:Yunnan | | - | | - | | FJ606554 | | **-** | | **-** | | ***-*** | | |
| *Lucanus cervus cervus* | **-** | | France:Basses Pyrenees | | - | | - | | FJ606555 | | **-** | | **-** | | ***-*** | | |
| *Lucanus kurosawai* | **-** | | Taiwan:Shinchu, Kuanwu | | - | | - | | FJ606556 | | **-** | | **-** | | ***-*** | | |
| *Lucanus kanoi piceus* | **-** | | Taiwan:Yilan, Shi-Yuan York | | - | | - | | FJ606557 | | **-** | | **-** | | ***-*** | | |
| *Lucanus kanoi kanoi* | **-** | | Taiwan:Nantou, Songkang | | - | | - | | FJ606558 | | **-** | | **-** | | ***-*** | | |
| *Lucanus fortunei* | **-** | | China:Fujian, San-Ming | | - | | - | | FJ606559 | | **-** | | **-** | | ***-*** | | |
| *Odontolabis sp.* | **-** | | Unknown | | - | | - | | KJ867474 | | **-** | | **-** | | ***-*** | | |
| *Platycerus caprea* | **-** | | Finland: Nylandia, Kirkkonummi, Sundsberg | | - | | - | | KJ963217 | | **-** | | **-** | | ***-*** | | |
| **Taxon** | **ID** | | **Collecting Locality** | | **GPS-latitude** | | **GPS-longitude** | | **COI** | | **16S rDNA** | | **28S rDNA** | | ***wingless*** | | |
| *Platycerus caprea* | **-** | | Finland: Northern Ostrobothnia, Ostrobottnia borealis pars australis | | **-** | | **-** | | KJ964717 | | **-** | | **-** | | ***-*** | | |
| *Platycerus caprea* | **-** | | Finland: Savonia australis, Ristiina, Montonen | | **-** | | **-** | | KJ965313 | | **-** | | **-** | | ***-*** | | |
| *Platycerus caprea* | **-** | | Estonia: Helme | | **-** | | **-** | | KJ966743 | | **-** | | **-** | | ***-*** | | |
| *Platycerus caprea* | **-** | | Russia: Uralskiy, Orenburg | | **-** | | **-** | | AB481434 | | **-** | | **-** | | ***-*** | | |
| *Platycerus caprea* | **-** | | Finland: Northern Ostrobothnia, Regio kuusamoensis | | **-** | | **-** | | HQ559246 | | **-** | | **-** | | ***-*** | | |
| *Platycerus virescens* | **-** | | USA: Illinois, Chicago, Palos Forest Reserve | | **-** | | **-** | | AB482186 | | **-** | | **-** | | ***-*** | | |
| *Platycerus hongwonpyoi* | **-** | | China: Hubei, Shennongjia | | **-** | | **-** | | AB482187 | | **-** | | **-** | | ***-*** | | |
| *Platycerus hongwonpyoi* | **-** | | China: Shaanxi, Zhouzhi Xian | | **-** | | **-** | | AB481423 | | **-** | | **-** | | ***-*** | | |
| *Platycerus hongwonpyoi* | **-** | | China: Shaanxi, Ningshan | | **-** | | **-** | | AB481427 | | **-** | | **-** | | ***-*** | | |
| *Platycerus caucasicus* | **-** | | Russia: Caucasus, Krasnodar, Tuapse | | **-** | | **-** | | AB482188 | | **-** | | **-** | | ***-*** | | |
| *Platycerus consimilis* | **-** | | China: Sichuan, Li Xian | | **-** | | **-** | | AB482189 | | **-** | | **-** | | ***-*** | | |
| *Platycerus sp.* | **-** | | China: Sichuan, Baoxing | | **-** | | **-** | | AB482190 | | **-** | | **-** | | ***-*** | | |
| *Platycerus hiurai* | **-** | | China: Sichuan | | **-** | | **-** | | AB482191 | | **-** | | **-** | | ***-*** | | |
| *Platycerus tieguanzi* | **-** | | China: Sichuan, Erlang Shan | | **-** | | **-** | | AB482192 | | **-** | | **-** | | ***-*** | | |
| *Platycerus tieguanzi* | **-** | | China: Sichuan, Emei-shan | | **-** | | **-** | | AB481425 | | **-** | | **-** | | ***-*** | | |
| *Platycerus turnai* | **-** | | China: Sichuan | | **-** | | **-** | | AB482193 | | **-** | | **-** | | ***-*** | | |
| *Platycerus feminatus* | **-** | | China: Sichuan, Ebian | | **-** | | **-** | | AB482194 | | **-** | | **-** | | ***-*** | | |
| *Platycerus caraboides* | **-** | | Ukraine: Kharkov | | **-** | | **-** | | AB481435 | | **-** | | **-** | | ***-*** | | |
| *Platycerus caraboides* | **-** | | Slovakia: Banska-Bystrica | | **-** | | **-** | | AB481436 | | **-** | | **-** | | ***-*** | | |
| *Platycerus caraboides* | **-** | | France: Paris | | **-** | | **-** | | AB481437 | | **-** | | **-** | | ***-*** | | |
| *Platycerus caraboides* | **-** | | Slovakia: Kisoce, Zlata Idka | | **-** | | **-** | | AB482195 | | **-** | | **-** | | ***-*** | | |
| *Platycerus caraboides* | **-** | | Germany: Rhineland Palatinate, Ahrtal, Ahrweiler | | **-** | | **-** | | KM446827 | | **-** | | **-** | | ***-*** | | |
| *Platycerus caraboides* | **-** | | Belgium: West-Vlaanderen, Vlanderen, Leuven, Blanden | | **-** | | **-** | | KM445575 | | **-** | | **-** | | ***-*** | | |
| *Platycerus oregonensis* | **-** | | USA: California | | **-** | | **-** | | AB482196 | | **-** | | **-** | | ***-*** | | |
| *Platycerus kawadai* | **-** | | Japan: Shizuoka, Misakubo | | **-** | | **-** | | AB481415 | | **-** | | **-** | | ***-*** | | |
| *Platycerus delicatulus* | **-** | | Japan: Yamagata, Oguni, Gomisawa, Hannari-daira | | **-** | | **-** | | AB481416 | | **-** | | **-** | | ***-*** | | |
| *Platycerus sugitai* | **-** | | Japan: Ehime, Ishiduchi-yama | | **-** | | **-** | | AB481417 | | **-** | | **-** | | ***-*** | | |
| *Platycerus urushiyamai* | **-** | | Japan: Oita, Makinoto-toge | | **-** | | **-** | | AB481418 | | **-** | | **-** | | ***-*** | | |
| *Platycerus akitaorum* | **-** | | Japan: Nara, Odaigahara | | **-** | | **-** | | AB481419 | | **-** | | **-** | | ***-*** | | |
| *Platycerus acuticollis* | **-** | | Japan: Osaka, Izumikatsuragisan | | **-** | | **-** | | AB481420 | | **-** | | **-** | | ***-*** | | |
| *Platycerus acuticollis* |  | | Japan: Fukuoka, Fukuchisan | | **-** | | **-** | | AB481421 | | **-** | | **-** | | ***-*** | | |
| *Platycerus acuticollis* | **-** | | Japan: Hyogo, Shiso, Hyonosen, Sakanotani | | **-** | | **-** | | AB481422 | | **-** | | **-** | | ***-*** | | |
| *Platycerus tabanai* | **-** | | China: Shaanxi, Zhouzhi Xian | | **-** | | **-** | | AB481424 | | **-** | | **-** | | ***-*** | | |
| *Platycerus rugosus* | **-** | | China: Chongqing, Daba Shan | | **-** | | **-** | | AB481429 | | **-** | | **-** | | ***-*** | | |
| *Platycerus businskyi* | **-** | | China: Shaanxi, Ningshan | | **-** | | **-** | | AB481430 | | **-** | | **-** | | ***-*** | | |
| **Taxon** | **ID** | | **Collecting Locality** | | **GPS-latitude** | | **GPS-longitude** | | **COI** | | **16S rDNA** | | **28S rDNA** | | ***wingless*** | | |
| *Platycerus dundai* | **-** | | China: Sichan, Gongga Shan | | **-** | | **-** | | AB481431 | | **-** | | **-** | | ***-*** | | |
| *Platycerus cupreimicans* | **-** | | China: Yunnan, Weixi | | **-** | | **-** | | AB481432 | | **-** | | **-** | | ***-*** | | |
| *Platycerus ladyae* | **-** | | China: Sichuan, Erlang Shan | | **-** | | **-** | | AB481433 | | **-** | | **-** | | ***-*** | | |
| *Platyceroides sp.* | **-** | | USA: California, Mendocino Co.Standish Hickey State Recreation | | **-** | | **-** | | AB482185 | | **-** | | **-** | | ***-*** | | |
| *Platyceropsis keeni* | **-** | | USA: Oregon, Lincoln Co., Woldport | | **-** | | **-** | | AB481426 | | **-** | | **-** | | ***-*** | | |
| *Prosopocoilus inclinatus inclinatus* | **-** | | Japan:Aomori, Kousei woodland pass | | **-** | | **-** | | AB110728 | | **-** | | **-** | | ***-*** | | |
| *Prosopocoilus inclinatus inclinatus* | **-** | | Japan:Aomori, Hirosaki, Ten-nouzawa | | **-** | | **-** | | AB110729 | | **-** | | **-** | | ***-*** | | |
| *Prosopocoilus inclinatus inclinatus* | **-** | | Japan:Aomori, Nishimeya, Meya dam | | **-** | | **-** | | AB110730 | | **-** | | **-** | | ***-*** | | |
| *Prosopocoilus astacoides blanchardi* | **-** | | Korea | | **-** | | **-** | | KF364622 | | **-** | | **-** | | ***-*** | | |
| *Prismognathus angularis angularis* | **-** | | Japan:Aomori, Nishimeya | | **-** | | **-** | | AB110731 | | **-** | | **-** | | ***-*** | | |
| *Prismognathus angularis angularis* | **-** | | Japan:Fukuoka, Soeda, Hikosan | | **-** | | **-** | | AB110732 | | **-** | | **-** | | ***-*** | | |
| *Prismognathus formosanus* | **-** | | Taiwan | | **-** | | **-** | | FJ606549 | | **-** | | **-** | | ***-*** | | |
| *Sinodendron cylindricum* | **-** | | Finland: Regio aboensis, Tammisaari, Riilahden kartano | | **-** | | **-** | | KJ966511 | | **-** | | **-** | | ***-*** | | |
| *Sinodendron cylindricum* | **-** | | Finland: Alandia, Finstroem, Attboele | | **-** | | **-** | | KJ961721 | | **-** | | **-** | | ***-*** | | |
| *Sinodendron cylindricum* | **-** | | Germany: Bavaria, Garmisch-Partenkirchen | | **-** | | **-** | | KM452001 | | **-** | | **-** | | ***-*** | | |
| *Aegus laevicollis subnitidus* | **-** | | Japan:Wakayama, Kiikatsuura | | **-** | | **-** | | - | | AB178301 | | **-** | | ***-*** | | |
| *Aesalus asiaticus asiaticus* | **-** | | Japan:Kyoto, Miyama, Ashu | | **-** | | **-** | | - | | AB178313 | | **-** | | ***-*** | | |
| *Ceruchus lignarius lignarius* | **-** | | Japan:Aomori, Hiraka, Mt. Ozaki | | **-** | | **-** | | - | | AB178311 | | **-** | | ***-*** | | |
| *Ceruchus lignarius* | **-** | | Japan:Mt. Fuji | | **-** | | **-** | | - | | AB178312 | | **-** | | ***-*** | | |
| *Colophon stokoei* | **-** | | South Africa: Hottentots Holland | | **-** | | **-** | | - | | KC763240 | | **-** | | ***-*** | | |
| *Colophon stokoei* | **-** | | South Africa: Hottentots Holland | | **-** | | **-** | | - | | KC763241 | | **-** | | ***-*** | | |
| *Colophon stokoei* | **-** | | South Africa: Hottentots Holland | | **-** | | **-** | | - | | KC763242 | | **-** | | ***-*** | | |
| *Colophon stokoei* | **-** | | South Africa: Hottentots Holland | | **-** | | **-** | | - | | KC763243 | | **-** | | ***-*** | | |
| *Colophon stokoei* | **-** | | South Africa: Hottentots Holland | | **-** | | **-** | | - | | KC763244 | | **-** | | ***-*** | | |
| *Colophon stokoei* | **-** | | South Africa: Hottentots Holland | | **-** | | **-** | | - | | KC763245 | | **-** | | ***-*** | | |
| *Colophon stokoei* | **-** | | South Africa: Palmiet River | | **-** | | **-** | | - | | KC763246 | | **-** | | ***-*** | | |
| *Colophon stokoei* | **-** | | South Africa: Palmiet River | | **-** | | **-** | | - | | KC763247 | | **-** | | ***-*** | | |
| *Colophon stokoei* | **-** | | South Africa: Palmiet River | | **-** | | **-** | | - | | KC763248 | | **-** | | ***-*** | | |
| *Colophon cameroni* | **-** | | South Africa: Hex River | | **-** | | **-** | | - | | KC763249 | | **-** | | ***-*** | | |
| *Colophon cameroni* | **-** | | South Africa: Hex River | | **-** | | **-** | | - | | KC763250 | | **-** | | ***-*** | | |
| *Colophon cameroni* | **-** | | South Africa: Hex River | | **-** | | **-** | | - | | KC763251 | | **-** | | ***-*** | | |
| *Colophon cameroni* | **-** | | South Africa: Hex River | | **-** | | **-** | | - | | KC763252 | | **-** | | ***-*** | | |
| *Colophon westwoodi* | **-** | | South Africa: Table Mountain | | **-** | | **-** | | - | | KC763253 | | **-** | | ***-*** | | |
| *Colophon westwoodi* | **-** | | South Africa: Table Mountain | | **-** | | **-** | | - | | KC763254 | | **-** | | ***-*** | | |
| *Colophon westwoodi* | **-** | | South Africa: Table Mountain | | **-** | | **-** | | - | | KC763255 | | **-** | | ***-*** | | |
| **Taxon** | **ID** | | **Collecting Locality** | | **GPS-latitude** | | **GPS-longitude** | | **COI** | | **16S rDNA** | | **28S rDNA** | | ***wingless*** | | |
| *Colophon haughtoni* | **-** | | South Africa: Hex River | | **-** | | **-** | | - | | KC763256 | | **-** | | ***-*** | | |
| *Colophon haughtoni* | **-** | | South Africa: Hex River | | **-** | | **-** | | - | | KC763257 | | **-** | | ***-*** | | |
| *Colophon haughtoni* | **-** | | South Africa: Hex River | | **-** | | **-** | | - | | KC763258 | | **-** | | ***-*** | | |
| *Colophon haughtoni* | **-** | | South Africa: Hex River | | **-** | | **-** | | - | | KC763259 | | **-** | | ***-*** | | |
| *Colophon izardi* | **-** | | South Africa: Langeberg | | **-** | | **-** | | - | | KC763260 | | **-** | | ***-*** | | |
| *Colophon izardi* | **-** | | South Africa: Langeberg | | **-** | | **-** | | - | | KC763261 | | **-** | | ***-*** | | |
| *Colophon izardi* | **-** | | South Africa: Langeberg | | **-** | | **-** | | - | | KC763262 | | **-** | | ***-*** | | |
| *Colophon izardi* | **-** | | South Africa: Langeberg | | **-** | | **-** | | - | | KC763263 | | **-** | | ***-*** | | |
| *Colophon sp.* | **-** | | South Africa: Swartberg | | **-** | | **-** | | - | | KC763264 | | **-** | | ***-*** | | |
| *Colophon sp.* | **-** | | South Africa: Swartberg | | **-** | | **-** | | - | | KC763265 | | **-** | | ***-*** | | |
| *Colophon neli* | **-** | | South Africa: Swartberg | | **-** | | **-** | | - | | KC763266 | | **-** | | ***-*** | | |
| *Colophon neli* | **-** | | South Africa: Swartberg | | **-** | | **-** | | - | | KC763267 | | **-** | | ***-*** | | |
| *Colophon neli* | **-** | | South Africa: Swartberg | | **-** | | **-** | | - | | KC763268 | | **-** | | ***-*** | | |
| *Colophon kawaii* | **-** | | South Africa: Hex River | | **-** | | **-** | | - | | KC763269 | | **-** | | ***-*** | | |
| *Colophon kawaii* | **-** | | South Africa: Hex River | | **-** | | **-** | | - | | KC763270 | | **-** | | ***-*** | | |
| *Colophon whitei* | **-** | | South Africa: Swartberg | | **-** | | **-** | | - | | KC763271 | | **-** | | ***-*** | | |
| *Colophon whitei* | **-** | | South Africa: Swartberg | | **-** | | **-** | | - | | KC763272 | | **-** | | ***-*** | | |
| *Colophon whitei* | **-** | | South Africa: Swartberg | | **-** | | **-** | | - | | KC763273 | | **-** | | ***-*** | | |
| *Colophon whitei* | **-** | | South Africa: Swartberg | | **-** | | **-** | | - | | KC763274 | | **-** | | ***-*** | | |
| *Colophon montisatris* | **-** | | South Africa: Swartberg | | **-** | | **-** | | - | | KC763275 | | **-** | | ***-*** | | |
| *Colophon montisatris* | **-** | | South Africa: Swartberg | | **-** | | **-** | | - | | KC763276 | | **-** | | ***-*** | | |
| *Colophon montisatris* | **-** | | South Africa: Swartberg | | **-** | | **-** | | - | | KC763277 | | **-** | | ***-*** | | |
| *Colophon eastmani nagaii* | **-** | | South Africa: Langeberg | | **-** | | **-** | | - | | KC763278 | | **-** | | ***-*** | | |
| *Colophon eastmani nagaii* | **-** | | South Africa: Langeberg | | **-** | | **-** | | - | | KC763281 | | **-** | | ***-*** | | |
| *Colophon eastmani nagaii* | **-** | | South Africa: Langeberg | | **-** | | **-** | | - | | KC763282 | | **-** | | ***-*** | | |
| *Colophon eastmani nagaii* | **-** | | South Africa: Langeberg | | **-** | | **-** | | - | | KC763283 | | **-** | | ***-*** | | |
| *Colophon eastmani nagaii* | **-** | | South Africa: Langeberg | | **-** | | **-** | | - | | KC763284 | | **-** | | ***-*** | | |
| *Colophon primosi* | **-** | | South Africa: Swartberg | | **-** | | **-** | | - | | KC763285 | | **-** | | ***-*** | | |
| *Colophon primosi* | **-** | | South Africa: Swartberg | | **-** | | **-** | | - | | KC763286 | | **-** | | ***-*** | | |
| *Colophon primosi* | **-** | | South Africa: Swartberg | | **-** | | **-** | | - | | KC763287 | | **-** | | ***-*** | | |
| *Colophon endroedyi* | **-** | | South Africa: Swartberg | | **-** | | **-** | | - | | KC763288 | | **-** | | ***-*** | | |
| *Colophon eastmani eastmani* | **-** | | South Africa: Langeberg | | **-** | | **-** | | - | | KC763279 | | **-** | | ***-*** | | |
| *Colophon eastmani eastmani* | **-** | | South Africa: Langeberg | | **-** | | **-** | | - | | KC763280 | | **-** | | ***-*** | | |
| *Dorcus titanus pilifer* | **-** | | Japan:Kyoto, Kyoto, Sakyo, Kyoto Univ. | | **-** | | **-** | | - | | AB178293 | | **-** | | ***-*** | | |
| *Dorcus titanus okinawanus* | **-** | | Japan:Okinawa, Okinawa I., Kunigami | | **-** | | **-** | | - | | AB178294 | | **-** | | ***-*** | | |
| **Taxon** | **ID** | | **Collecting Locality** | | **GPS-latitude** | | **GPS-longitude** | | **COI** | | **16S rDNA** | | **28S rDNA** | | ***wingless*** | | |
| *Dorcus japonicus* | **-** | | Japan:Kagoshima, Tokuno Island, Isen | | **-** | | **-** | | **-** | | AB236863 | | **-** | | ***-*** | | |
| *Dorcus carinulatus* | **-** | | Taiwan:Taichung, Fengyuan | | **-** | | **-** | | **-** | | AB236864 | | **-** | | ***-*** | | |
| *Dorcus taiwanicus* | **-** | | Taiwan:Nantou, Habonshan | | **-** | | **-** | | **-** | | AB236865 | | **-** | | ***-*** | | |
| *Dorcus velutinus* | **-** | | Thailand:Chiang Mai, Doi Saket | | **-** | | **-** | | **-** | | AB236866 | | **-** | | ***-*** | | |
| *Dorcus curvidens binodulosus* | **-** | | Japan:Yamanashi | | **-** | | **-** | | **-** | | AB178292 | | **-** | | ***-*** | | |
| *Dorcus rectus rectus* | **-** | | Japan:Aomori, Nishimeya, Meya dam | | **-** | | **-** | | **-** | | AB178295 | | **-** | | ***-*** | | |
| *Figulus binodulus* | **-** | | Japan:Kyoto, Kyoto, Sakyo, Mt. Yoshida | | **-** | | **-** | | **-** | | AB178308 | | **-** | | ***-*** | | |
| *Figulus boninensis* | **-** | | Japan:Tokyo, Ogasawara Is., Chichizima I., Mt. Tsutsuzi | | **-** | | **-** | | **-** | | AB178309 | | **-** | | ***-*** | | |
| *Lucanus maculifemoratus maculifemoratus* | **-** | | Japan:Aomori, Nishimeya, Meya dam | | **-** | | **-** | | **-** | | AB178302 | | **-** | | ***-*** | | |
| *Lucanus maculifemoratus* | **-** | | Unknown | | **-** | | **-** | | **-** | | JQ066817 | | **-** | | ***-*** | | |
| *Lucanus ferriei* | **-** | | Japan:Kagoshima, Amami I. | | **-** | | **-** | | **-** | | AB178303 | | **-** | | ***-*** | | |
| *Neolucanus protogenetivus protogenetivus* | **-** | | Japan:Kagoshima, Amami I., Mt. Yui | | **-** | | **-** | | **-** | | AB178304 | | **-** | | ***-*** | | |
| *Neolucanus protogenetivus hamaii* | **-** | | Japan:Kagoshima, Uke I., Daisen | | **-** | | **-** | | **-** | | AB178305 | | **-** | | ***-*** | | |
| *Nicagus japonicus* | **-** | | Japan:Aomori, Fukaura, Oirase | | **-** | | **-** | | **-** | | AB178314 | | **-** | | ***-*** | | |
| *Nigidius lewisi* | **-** | | Japan:Wakayama, Kiikatsuura | | **-** | | **-** | | **-** | | AB178310 | | **-** | | ***-*** | | |
| *Nigidius delegorguei* | **-** | | South Africa: KwazuluNatal, Ithala Game Reserve | | **-** | | **-** | | **-** | | JN969133 | | **-** | | ***-*** | | |
| *Platycerus hongwonpyoi* | **-** | | South Korea: Jiri-san | | **-** | | **-** | | **-** | | AB490698 | | **-** | | ***-*** | | |
| *Platycerus hongwonpyoi* | **-** | | South Korea: Kaji-san | | **-** | | **-** | | **-** | | AB490699 | | **-** | | ***-*** | | |
| *Platycerus hongwonpyoi* | **-** | | South Korea: Odae-san | | **-** | | **-** | | **-** | | AB490700 | | **-** | | ***-*** | | |
| *Platycerus hongwonpyoi* | **-** | | China: Hubei, Shennonja | | **-** | | **-** | | **-** | | AB490704 | | **-** | | ***-*** | | |
| *Platycerus hongwonpyoi* | **-** | | China: Shaanxi, Zhouzhi Xian | | **-** | | **-** | | **-** | | AB489979 | | **-** | | ***-*** | | |
| *Platycerus hongwonpyoi* | **-** | | China: Shaanxi, Ningshan | | **-** | | **-** | | **-** | | AB489985 | | **-** | | ***-*** | | |
| *Platycerus caraboides* | **-** | | Ukraine: Kharkov | | **-** | | **-** | | **-** | | AB490166 | | **-** | | ***-*** | | |
| *Platycerus caraboides* | **-** | | Slovakia: Banska-Bystrica | | **-** | | **-** | | **-** | | AB490167 | | **-** | | ***-*** | | |
| *Platycerus caraboides* | **-** | | France: Paris | | **-** | | **-** | | **-** | | AB490168 | | **-** | | ***-*** | | |
| *Platycerus caraboides* | **-** | | Slovakia: Kosice, Zlata Idka | | **-** | | **-** | | **-** | | AB490169 | | **-** | | ***-*** | | |
| *Platycerus rugosus* | **-** | | China: Chongqing, Daba Shan | | **-** | | **-** | | **-** | | AB489980 | | **-** | | ***-*** | | |
| *Platycerus rugosus* | **-** | | China: Sichuan, Nanjiang, Mican-Shan | | **-** | | **-** | | **-** | | AB490701 | | **-** | | ***-*** | | |
| *Platycerus rugosus* | **-** | | China: Sichuan, Nanjiang, Mican-Shan | | **-** | | **-** | | **-** | | AB490702 | | **-** | | ***-*** | | |
| *Platycerus rugosus* | **-** | | China: Hubei, Shennonja | | **-** | | **-** | | **-** | | AB490703 | | **-** | | ***-*** | | |
| *Platycerus consimilis* | **-** | | China: Sichuan, Nanjiang, Mican-Shan | | **-** | | **-** | | **-** | | AB490694 | | **-** | | ***-*** | | |
| *Platycerus consimilis* | **-** | | China: Sichuan, Mialuo | | **-** | | **-** | | **-** | | AB490695 | | **-** | | ***-*** | | |
| *Platycerus acuticollis acuticollis* | **-** | | Japan:Niigata, Joetsu, Mt. Nanba | | **-** | | **-** | | **-** | | AB178307 | | **-** | | ***-*** | | |
| *Platycerus acuticollis* | **-** | | Japan: Gunma, Hoshi-Onsen | | **-** | | **-** | | **-** | | AB490400 | | **-** | | ***-*** | | |
| *Platycerus bashanicus* | **-** | | China: Chongqing, Guangto-Shan | | **-** | | **-** | | **-** | | AB490296 | | **-** | | ***-*** | | |
| **Taxon** | **ID** | | **Collecting Locality** | | **GPS-latitude** | | **GPS-longitude** | | **COI** | | **16S rDNA** | | **28S rDNA** | | ***wingless*** | | |
| *Platycerus bashanicus* | **-** | | China: Chongqing, Guangto-Shan | | **-** | | **-** | | **-** | | AB490297 | | **-** | | ***-*** | | |
| *Platycerus feminatus* | **-** | | China: Sichuan, Ebian | | **-** | | **-** | | **-** | | AB489986 | | **-** | | ***-*** | | |
| *Platycerus feminatus* | **-** | | China: Sichuan, Meigu | | **-** | | **-** | | **-** | | AB490696 | | **-** | | ***-*** | | |
| *Platycerus turnai* | **-** | | China: Sichuan | | **-** | | **-** | | **-** | | AB490300 | | **-** | | ***-*** | | |
| *Platycerus turnai* | **-** | | China: Hubei, Shennonja | | **-** | | **-** | | **-** | | AB490697 | | **-** | | ***-*** | | |
| *Platycerus delicatulus delicatulus* | **-** | | Japan:Aomori, Towadako, Tsuta | | **-** | | **-** | | **-** | | AB178306 | | **-** | | ***-*** | | |
| *Platycerus delicatulus* | **-** | | Japan: Yamagata, Oguni, Gomizawa | | **-** | | **-** | | **-** | | AB490402 | | **-** | | ***-*** | | |
| *Platycerus businskyi* | **-** | | China: Shaanxi, Ningshan | | **-** | | **-** | | **-** | | AB489981 | | **-** | | ***-*** | | |
| *Platycerus tabanai* | **-** | | China: Shaanxi, Zhouzhi Xian | | **-** | | **-** | | **-** | | AB489982 | | **-** | | ***-*** | | |
| *Platycerus dundai* | **-** | | China: Sichuan, Gongga shan | | **-** | | **-** | | **-** | | AB489983 | | **-** | | ***-*** | | |
| *Platycerus cupreimicans* | **-** | | China: Yunnan, Weixi | | **-** | | **-** | | **-** | | AB489984 | | **-** | | ***-*** | | |
| *Platycerus hiurai* | **-** | | China: Sichuan | | **-** | | **-** | | **-** | | AB489987 | | **-** | | ***-*** | | |
| *Platycerus sp.* |  | | China: Sichuan, Baoxing | | **-** | | **-** | | **-** | | AB489988 | | **-** | | ***-*** | | |
| *Platycerus caprea* | **-** | | Russia: Uralskiy, Orenburg | | **-** | | **-** | | **-** | | AB490165 | | **-** | | ***-*** | | |
| *Platycerus oregonensis* | **-** | | USA: California | | **-** | | **-** | | **-** | | AB490170 | | **-** | | ***-*** | | |
| *Platycerus virescens* | **-** | | USA: Illinois, Chicago, Palos Forest Reserve | | **-** | | **-** | | **-** | | AB490171 | | **-** | | ***-*** | | |
| *Platycerus caucasicus* | **-** | | Russia: Caucasus, Krasnodar, Tuapse | | **-** | | **-** | | **-** | | AB490172 | | **-** | | ***-*** | | |
| *Platycerus consimilis* | **-** | | China: Sichuan, Li Xian | | **-** | | **-** | | **-** | | AB490173 | | **-** | | ***-*** | | |
| *Platycerus tieguanzi* | **-** | | China: Sichuan, Erlang Shan | | **-** | | **-** | | **-** | | AB490174 | | **-** | | ***-*** | | |
| *Platycerus nagahatai* | **-** | | China: Shaanxi, Zhouzhi Xian | | **-** | | **-** | | **-** | | AB490293 | | **-** | | ***-*** | | |
| *Platycerus yeren* | **-** | | China: Hubei, Shennonja | | **-** | | **-** | | **-** | | AB490294 | | **-** | | ***-*** | | |
| *Platycerus kitawakii* | **-** | | China: Chongqing, Daba Shan | | **-** | | **-** | | **-** | | AB490295 | | **-** | | ***-*** | | |
| *Platycerus tangi* | **-** | | China: Sichuan, MaoXian | | **-** | | **-** | | **-** | | AB490298 | | **-** | | ***-*** | | |
| *Platycerus cyanidraconis* | **-** | | China: Sichuan, Mao Xian | | **-** | | **-** | | **-** | | AB490299 | | **-** | | ***-*** | | |
| *Platycerus ladyae* | **-** | | China: Sichuan, Erlang Shan | | **-** | | **-** | | **-** | | AB490301 | | **-** | | ***-*** | | |
| *Platycerus albisomni* | **-** | | Japan: Yamagata, Nishikawa, Gassan, Shizu | | **-** | | **-** | | **-** | | AB490401 | | **-** | | ***-*** | | |
| *Platycerus akitaorum* | **-** | | Japan: Nara, Odaigahara | | **-** | | **-** | | **-** | | AB490405 | | **-** | | ***-*** | | |
| *Platycerus kawadai* | **-** | | Japan: Shizuoka, Misakubo | | **-** | | **-** | | **-** | | AB490403 | | **-** | | ***-*** | | |
| *Platycerus sugitai* | **-** | | Japan: Ehime, Ishizuchi-yama | | **-** | | **-** | | **-** | | AB490404 | | **-** | | ***-*** | | |
| *Platycerus takakuwai* | **-** | | Japan: Yamanashi, Enzan, Daibosatsu | | **-** | | **-** | | **-** | | AB490406 | | **-** | | ***-*** | | |
| *Platycerus sue* | **-** | | Japan: Ehime, Ishizuchi-yama | | **-** | | **-** | | **-** | | AB490407 | | **-** | | ***-*** | | |
| *Platycerus viridicuprus* | **-** | | Japan: Hyogo, Shiso, Hyonosen, Sakanotani | | **-** | | **-** | | **-** | | AB490408 | | **-** | | ***-*** | | |
| *Platycerus urushiyamai* | **-** | | Japan: Oita, Kuju-san, Makinoto-toge | | **-** | | **-** | | **-** | | AB490409 | | **-** | | ***-*** | | |
| *Platycerus primigenius* | **-** | | Russia: Krasnodar, Azish-Tau | | **-** | | **-** | | **-** | | AB490779 | | **-** | | ***-*** | | |
| *Platyceroides sp.* | **-** | | USA: California, Mendocino | | **-** | | **-** | | **-** | | AB490780 | | **-** | | ***-*** | | |
| **Taxon** | **ID** | | **Collecting Locality** | | **GPS-latitude** | | **GPS-longitude** | | **COI** | | **16S rDNA** | | **28S rDNA** | | ***wingless*** | | |
| *Platyceropsis keeni* | **-** | | USA: Oregon, Lincoln Co., Woldport | | **-** | | **-** | | **-** | | AB490781 | | **-** | | ***-*** | | |
| *Prismognathus angularis angularis* | **-** | | Japan:Fukuoka, Mt. Hikosan | | **-** | | **-** | | **-** | | AB178299 | | **-** | | ***-*** | | |
| *Prismognathus dauricus* | **-** | | Japan:Nagasaki, Tsushima, Kamiagata | | **-** | | **-** | | **-** | | AB178300 | | **-** | | ***-*** | | |
| *Prosopocoilus inclinatus inclinatus* | **-** | | Japan:Aomori, Nishimeya, Meya dam | | **-** | | **-** | | **-** | | AB178297 | | **-** | | ***-*** | | |
| *Prosopocoilus pseudodissimilis* | **-** | | Japan:Okinawa, Ishigaki I. | | **-** | | **-** | | **-** | | AB178298 | | **-** | | ***-*** | | |
| *Rhaetulus recticornis* | **-** | | Japan:Kagoshima, Amami I. | | **-** | | **-** | | **-** | | AB178296 | | **-** | | ***-*** | | |
| *Aegus laevicollis* | **-** | | Unknown | | **-** | | **-** | | **-** | | - | | KP250386 | | ***-*** | | |
| *Aegus parallelus* | **-** | | Unknown | | **-** | | **-** | | **-** | | - | | KP250387 | | ***-*** | | |
| *Aesalus scarabaeoides* | **-** | | Unknown | | **-** | | **-** | | **-** | | - | | KP250388 | | ***-*** | | |
| *Apterocyclus honoluluensis* | **-** | | Unknown | | **-** | | **-** | | **-** | | - | | KP250389 | | ***-*** | | |
| *Auxicerus platyceps* | **-** | | Unknown | | **-** | | **-** | | **-** | | - | | KP250390 | | ***-*** | | |
| *Calcodes aeratus* | **-** | | Unknown | | **-** | | **-** | | **-** | | - | | KP250392 | | ***-*** | | |
| *Casignetus spixi* | **-** | | Unknown | | **-** | | **-** | | **-** | | - | | KP250393 | | ***-*** | | |
| *Cantharolethrus steinheili* | **-** | | Unknown | | **-** | | **-** | | **-** | | - | | KP250394 | | ***-*** | | |
| *Ceruchus chrysomelinus* | **-** | | Unknown | | **-** | | **-** | | **-** | | - | | KP250395 | | ***-*** | | |
| *Ceruchus piceus* | **-** | | Unknown | | **-** | | **-** | | **-** | | - | | KP250396 | | ***-*** | | |
| *Cyclommatus alagari* | **-** | | Unknown | | **-** | | **-** | | **-** | | - | | KP250406 | | ***-*** | | |
| *Cyclommatus metallifer* | **-** | | Unknown | | **-** | | **-** | | **-** | | - | | KP250407 | | ***-*** | | |
| *Colophon cameroni* | **-** | | Unknown | | **-** | | **-** | | **-** | | - | | KP250401 | | ***-*** | | |
| *Colophon eastmani* | **-** | | Unknown | | **-** | | **-** | | **-** | | - | | KP250402 | | ***-*** | | |
| *Colophon montisatris* | **-** | | Unknown | | **-** | | **-** | | **-** | | - | | KP250403 | | ***-*** | | |
| *Colophon stokoei* | **-** | | Unknown | | **-** | | **-** | | **-** | | - | | KP250404 | | ***-*** | | |
| *Colophon westwoodi* | **-** | | Unknown | | **-** | | **-** | | **-** | | - | | KP250405 | | ***-*** | | |
| *Chiasognathus grantii* | **-** | | Unknown | | **-** | | **-** | | **-** | | - | | KP250397 | | ***-*** | | |
| *Chiasognathus mniszechii* | **-** | | Unknown | | **-** | | **-** | | **-** | | - | | KP250398 | | ***-*** | | |
| *Chiasognathus latreillei* | **-** | | Unknown | | **-** | | **-** | | **-** | | - | | KP250399 | | ***-*** | | |
| *Chiasognathus sombrus* | **-** | | Unknown | | **-** | | **-** | | **-** | | - | | KP250400 | | ***-*** | | |
| *Chileistomus cucullatus* | **-** | | Unknown | | **-** | | **-** | | **-** | | - | | KP250438 | | ***-*** | | |
| *Dorcus parallelus* | **-** | | Unknown | | **-** | | **-** | | **-** | | - | | KP250410 | | ***-*** | | |
| *Dorcus rectus* | **-** | | Unknown | | **-** | | **-** | | **-** | | - | | KP250411 | | ***-*** | | |
| *Dorcus rubrofemoratus* | **-** | | Unknown | | **-** | | **-** | | **-** | | - | | KP250412 | | ***-*** | | |
| *Dorcus titanus* | **-** | | Unknown | | **-** | | **-** | | **-** | | - | | KP250413 | | ***-*** | | |
| *Dorculus lombokensis* | **-** | | Unknown | | **-** | | **-** | | **-** | | - | | KP250409 | | ***-*** | | |
| *Eucarteria subvittata* | **-** | | Unknown | | **-** | | **-** | | **-** | | - | | KP250414 | | ***-*** | | |
| *Hexarthrius mandibularis* | **-** | | Unknown | | **-** | | **-** | | **-** | | - | | KP250417 | | ***-*** | | |
| **Taxon** | **ID** | | **Collecting Locality** | | **GPS-latitude** | | **GPS-longitude** | | **COI** | | **16S rDNA** | | **28S rDNA** | | ***wingless*** | | |
| *Lamprima adolphinae* | **-** | | Unknown | | **-** | | **-** | | **-** | | - | | KP250418 | | ***-*** | | |
| *Lamprima aenea* | **-** | | Unknown | | **-** | | **-** | | **-** | | - | | KP250419 | | ***-*** | | |
| *Lamprima insularis* | **-** | | Unknown | | **-** | | **-** | | **-** | | - | | KP250420 | | ***-*** | | |
| *Lamprima latreillii* | **-** | | Unknown | | **-** | | **-** | | **-** | | - | | KP250421 | | ***-*** | | |
| *Lissotes rudis* | **-** | | Unknown | | **-** | | **-** | | **-** | | - | | KP250422 | | ***-*** | | |
| *Lucanus laetus* | **-** | | Unknown | | **-** | | **-** | | **-** | | - | | KP250423 | | ***-*** | | |
| *Lucanus mazama* | **-** | | Unknown | | **-** | | **-** | | **-** | | - | | KP250424 | | ***-*** | | |
| *Lucanus treadon* | **-** | | Unknown | | **-** | | **-** | | **-** | | - | | JN969226 | | ***-*** | | |
| *Lucanus sp.* | **-** | | Unknown | | **-** | | **-** | | **-** | | - | | KJ845137 | | ***-*** | | |
| *Mesotopus regius* | **-** | | Unknown | | **-** | | **-** | | **-** | | - | | KP250425 | | ***-*** | | |
| *Nicagus obscurus* | **-** | | Unknown | | **-** | | **-** | | **-** | | - | | KJ845143 | | ***-*** | | |
| *Nigidius sp.* | **-** | | Unknown | | **-** | | **-** | | **-** | | - | | KP250427 | | ***-*** | | |
| *Nigidius delegorguei* | **-** | | South Africa: KwazuluNatal, Ithala Game Reserve | | **-** | | **-** | | **-** | | - | | JN969227 | | ***-*** | | |
| *Noseolucanus rugosus* | **-** | | Unknown | | **-** | | **-** | | **-** | | - | | KP250426 | | ***-*** | | |
| *Odontolabis cuvera* | **-** | | Unknown | | **-** | | **-** | | **-** | | - | | KP250429 | | ***-*** | | |
| *Phalacrognathus muelleri* | **-** | | Unknown | | **-** | | **-** | | **-** | | - | | KP250432 | | ***-*** | | |
| *Prosopocoilus girafa* | **-** | | Unknown | | **-** | | **-** | | **-** | | - | | KP250433 | | ***-*** | | |
| *Prosopocoilus natalensis* | **-** | | Unknown | | **-** | | **-** | | **-** | | - | | KP250434 | | ***-*** | | |
| *Prosopocoilus savagei* | **-** | | Unknown | | **-** | | **-** | | **-** | | - | | KP250435 | | ***-*** | | |
| *Ryssonotus laticeps* | **-** | | Unknown | | **-** | | **-** | | **-** | | - | | KP250436 | | ***-*** | | |
| *Ryssonotus nebulosus* | **-** | | Unknown | | **-** | | **-** | | **-** | | - | | KP250437 | | ***-*** | | |
| *Sinodendron cylindricum* | **-** | | Unknown | | **-** | | **-** | | **-** | | - | | KP250440 | | ***-*** | | |
| *Sinodendron rugosum* | **-** | | Unknown | | **-** | | **-** | | **-** | | - | | KP250441 | | ***-*** | | |
| *Sphaenognathus alticollis* | **-** | | Unknown | | **-** | | **-** | | **-** | | - | | KP250442 | | ***-*** | | |
| *Sphaenognathus giganteus* | **-** | | Unknown | | **-** | | **-** | | **-** | | - | | KP250443 | | ***-*** | | |
| *Sphaenognathus kolbei* | **-** | | Unknown | | **-** | | **-** | | **-** | | - | | KP250444 | | ***-*** | | |
| *Sphaenognathus monguilloni* | **-** | | Unknown | | **-** | | **-** | | **-** | | - | | KP250445 | | ***-*** | | |
| *Sphaenognathus munchowae* | **-** | | Unknown | | **-** | | **-** | | **-** | | - | | KP250446 | | ***-*** | | |
| *Sphaenognathus nobilis* | **-** | | Unknown | | **-** | | **-** | | **-** | | - | | KP250447 | | ***-*** | | |
| *Sphaenognathus queenslandicus* | **-** | | Unknown | | **-** | | **-** | | **-** | | - | | KP250448 | | ***-*** | | |
| *Sphaenognathus taschenbergi* | **-** | | Unknown | | **-** | | **-** | | **-** | | - | | KP250449 | | ***-*** | | |
| *Sphaenognathus xerophilus* | **-** | | Unknown | | **-** | | **-** | | **-** | | - | | KP250450 | | ***-*** | | |
| *Streptocerus speciosus* | **-** | | Unknown | | **-** | | **-** | | **-** | | - | | KP250451 | | ***-*** | | |
| *Weinreichius perroti* | **-** | | Unknown | | **-** | | **-** | | **-** | | - | | KP250452 | | ***-*** | | |
| *Yumikoi makii* | **-** | | Unknown | | **-** | | **-** | | **-** | | - | | KP250453 | | ***-*** | | |
