# Supplementary material for: Subspecific Differentiation Events of Montane Stag Beetles (Coleoptera, Lucanidae) Endemic to Formosa Island
Source: PLoS One. 2016 Jun 3;11(6):e0156600. doi: 10.1371/journal.pone.0156600 (PMC4892689; doi:10.1371/journal.pone.0156600)
Supplement: S2 Table — (DOC) [file pone.0156600.s003.doc]

S2 Table. Heterogeneous positions detected in *wingless* sequence chromatogram among *Lucanus* *kanoi*, *L*. *maculifemoratus* *taiwanus*, and *L*. *ogakii*

|  | **Sample** | **Position** | | | | | | | | | | |
| --- | --- | --- | --- | --- | --- | --- | --- | --- | --- | --- | --- | --- |
|  |  | **34** | **43** | **58** | **100** | **106** | **181** | **199** | **247** | **301** | **310** | **331** |
| ***L*. *k*. *kanoi*** | **Luc544** | G | C | C | K | Y | G | C | T | C | C | C |
|  | **Luc545** | G | C | C | G | T | G | C | C | C | C | C |
|  | **Luc555** | G | C | C | G | Y | G | C | T | C | C | C |
| ***L*. *k*. *piceus*** | **Luc741** | A | C | C | T | T | G | Y | T | C | C | C |
|  | **Luc1095** | R | S | C | K | Y | G | Y | T | C | C | C |
|  | **Luc1096** | A | C | C | T | T | G | Y | T | C | C | C |
|  | **Luc1097** | G | G | C | G | C | G | C | T | C | C | C |
|  | **Luc1098** | G | C | C | G | C | G | C | T | C | C | C |
| ***L*. *m*. *taiwanus*** | **Luc112** | G | C | C | T | C | R | C | T | C | C | C |
|  | **Luc188** | G | C | C | G | T | A | C | Y | C | C | C |
|  | **Luc189** | G | C | C | G | C | G | C | C | C | T | T |
|  | **Luc193** | G | C | T | G | T | A | C | Y | C | T | T |
|  | **Luc194** | G | C | C | G | Y | R | C | T | C | C | C |
|  | **Luc197** | G | C | C | G | Y | A | C | T | C | C | C |
|  | **Luc198** | G | C | C | K | Y | A | C | Y | C | Y | Y |
|  | **Luc207** | G | C | C | G | Y | G | C | C | C | Y | C |
|  | **Luc498** | G | C | Y | G | Y | R | Y | T | C | C | C |
|  | **Luc499** | G | C | C | G | Y | A | C | T | Y | Y | C |
|  | **Luc500** | G | C | C | G | C | G | C | C | C | T | T |
|  | **Luc501** | G | C | C | G | T | A | C | T | C | Y | C |
|  | **Luc502** | G | C | T | G | T | A | C | T | Y | Y | C |
|  | **Luc562** | G | C | C | G | T | A | C | Y | C | T | T |
|  | **Luc787** | G | C | C | G | C | G | C | C | C | T | T |
|  | **Luc1102** | G | C | T | G | T | A | C | T | C | C | C |
| ***L*. *o*. *ogakii*** | **Luc111** | G | C | C | G | C | G | C | T | C | C | C |
|  | **Luc135** | G | C | C | G | C | G | Y | Y | C | Y | C |
|  | **Luc200** | G | C | C | G | C | G | Y | C | C | Y | C |
|  | **Luc1100** | G | C | C | G | C | G | C | T | C | C | C |
| ***L*. *o*. *chuyunshanus*** | **Luc542** | G | C | C | K | C | G | C | C | C | Y | Y |
|  | **Luc543** | G | C | C | G | C | G | C | T | C | C | C |
|  | **Luc1101** | G | C | C | G | Y | A | C | T | C | C | C |
