# Supplementary material for: Subspecific Differentiation Events of Montane Stag Beetles (Coleoptera, Lucanidae) Endemic to Formosa Island
Source: PLoS One. 2016 Jun 3;11(6):e0156600. doi: 10.1371/journal.pone.0156600 (PMC4892689; doi:10.1371/journal.pone.0156600)
Supplement: S3 Table — (DOC) [file pone.0156600.s004.doc]

S3 Table. Heterogeneous positions detected in *wingless* sequence chromatogram between *Pri*. *davidis cheni* and *Pri*. *d*. *nigerrimus* and that between *Pri*. *formosanus* and *Pri*. *piluensis*

| **Taxa** | **Sample** | **Position** | | | |
| --- | --- | --- | --- | --- | --- |
|  |  | **72** | **221** | **269** | **371** |
| ***Pri*. *d*. *cheni*** | **Luc554** | **-** | **-** | G | C |
|  | **Luc1290** | **-** | **-** | G | C |
|  | **Luc1292** | **-** | **-** | G | C |
| ***Pri*. *d*. *nigerrimus*** | **Luc245** | **-** | **-** | C | S |
|  | **Luc726** | **-** | **-** | C | S |
|  | **Luc727** | **-** | **-** | C | S |
|  | **Luc1066** | **-** | **-** | S | C |
| ***Pri*. *formosanus*** | **Luc102** | C | G | **-** | **-** |
|  | **Luc728** | C | G | **-** | **-** |
|  | **Luc1073** | M | G | **-** | **-** |
| ***Pri*. *piluensis*** | **Luc472** | A | G | **-** | **-** |
|  | **Luc558** | A | S | **-** | **-** |
|  | **Luc559** | M | G | **-** | **-** |
|  | **Luc1297** | A | G | **-** | **-** |
|  | **Luc1298** | A | G | **-** | **-** |
